# Comprehensive Insights into Sarcopenia in Dialysis Patients: Mechanisms, Assessment, and Therapeutic Approaches

**DOI:** 10.3390/medicina61030449

**Published:** 2025-03-04

**Authors:** Mariateresa Zicarelli, Anila Duni, Konstantinos Leivaditis, Yu-Li Lin, Federica Baciga, Sara Pugliese, Marco Fiorentino, Bang-Gee Hsu, Stefanos Roumeliotis, Yuri Battaglia, Evangelia Dounousi, Davide Bolignano

**Affiliations:** 1 Department of Health Sciences, University “Magna-Graecia” of Catanzaro, 88100 Catanzaro, Italy; 2 2nd Department of Nephrology, Faculty of Medicine, School of Health Sciences, University of Ioannina, 45110 Ioannina, Greece; 3 2nd Department of Nephrology, AHEPA Hospital, School of Medicine, Aristotle University of Thessaloniki, 54124 Thessaloniki, Greece; 4 Division of Nephrology, Hualien Tzu Chi Hospital, Buddhist Tzu Chi Medical Foundation, Hualien 970473, Taiwan; 5 School of Medicine, Tzu Chi University, Hualien 970473, Taiwan; 6 Department of Medicine, University of Verona, 37129 Verona, Italy; 7 Nephrology and Dialysis Unit, Pederzoli Hospital, Peschiera del Garda, 37129 Verona, Italy; 8 School of Medicine, University “Magna-Graecia” of Catanzaro, 88100 Catanzaro, Italy; 9 Nephrology, Dialysis and Transplantation Unit, Department of Precision and Regenerative Medicine and Ionian Area, University of Bari Aldo Moro, 70121 Bari, Italy; 10 Department of Medical and Surgical Sciences, University “Magna-Graecia” of Catanzaro, 88100 Catanzaro, Italy

**Keywords:** sarcopenia, end-stage kidney disease, hemodialysis, peritoneal dialysis

## Abstract

Sarcopenia, defined as the progressive loss of muscle mass, strength, and function, is largely prevalent but still clinically underrecognized among patients undergoing chronic dialysis therapy. The pathogenesis involves a complex interplay of chronic inflammation, oxidative stress, metabolic acidosis, hormonal imbalances, protein waste, malnutrition, and reduced physical activity. This multifactorial condition profoundly impairs quality of life and may lead to significant clinical consequences, including frailty, an increased risk of falls and hospitalization, and elevated mortality. Despite its clinical relevance, sarcopenia often remains underdiagnosed due to inconsistent diagnostic criteria and challenges in assessing body composition in dialysis populations. Therapeutic strategies, including tailored exercise programs, nutritional interventions, and pharmacological treatments, are essential to mitigate muscle loss and improve patient outcomes. Early identification and routine sarcopenia assessment in clinical practice could play a pivotal role in enhancing the management of dialysis patients. A multidisciplinary, personalized approach is necessary to address the diverse factors contributing to sarcopenia and to improve the overall prognosis and quality of life for this vulnerable population.

## 1. Introduction

Chronic kidney disease (CKD) represents a growing global health challenge, with its prevalence rising steadily over recent decades [1]. Approximately 10% of the worldwide population is estimated to suffer from some degree of CKD, and many of these patients ultimately progress to end-stage kidney disease (ESKD), requiring renal replacement therapy for a lifetime [1]. Chronic dialysis remains the most employed renal replacement modality, with millions of individuals worldwide depending on hemodialysis (HD) or peritoneal dialysis (PD) for survival. However, the burden of dialysis extends far beyond its technical demands, imposing substantial economic, societal, and personal costs due to cardiovascular complications, more frequent hospitalizations, and severely impaired physical functioning, all negatively affecting the quality of life and well-being [2,3,4].

Sarcopenia, a condition characterized by the progressive loss of muscle mass, strength, and function, is largely prevalent among dialysis patients [5]. While traditionally considered an aging-associated condition, sarcopenia is now increasingly recognized as a significant issue in younger populations with chronic illnesses [6], including ESKD. This condition exacerbates many of the challenges already faced by dialysis patients, such as physical disability, depression, and an increased risk of falls and hospitalizations. Sarcopenia also portends worse clinical outcomes, including higher mortality, underscoring its importance as a clinical entity [7].

Despite its profound impact, sarcopenia remains underdiagnosed and clinically underestimated in the dialysis population. However, emerging evidence suggests that sarcopenia is not merely a consequence of ESKD but also a modifiable risk factor that warrants greater attention.

In this review, we will explore the multifaceted relationship between sarcopenia and dialysis, focusing on its epidemiology, pathophysiology, and clinical implications through a pragmatic, expert-driven literature selection approach. We will discuss the unique mechanisms driving sarcopenia in this population and address the challenges in diagnosing sarcopenia. Finally, we will propose early recognition and therapeutic strategies, emphasizing the importance of incorporating sarcopenia assessment into routine care for dialysis patients.

This review is not meant as a clinical practice guideline for diagnostic or therapeutic decision making in patients with sarcopenia and dialysis. Instead, its objective is to provide a comprehensive overview of the current state of knowledge, synthesizing key findings from the literature to enhance understanding of the underlying mechanisms, assessment methods, and clinical implications of sarcopenia in this specific population setting.

## 2. Epidemiology of Sarcopenia in Dialysis Patients

Studies from the general population report that the prevalence of sarcopenia in healthy adults older than 60 years is around 10%, and it substantially increases among patients with CKD [8,9]. In people with ESKD requiring chronic dialysis, the reported prevalence ranges from 1.5% to 68% [10,11]. The high variation in prevalence might be partly ascribed to heterogenous study design and differences in the patient populations’ characteristics related to age, gender, or ethnicity and, above all, to the dialysis modality itself (PD vs. HD) [10,11,12]. Furthermore, despite the established general agreement that the definition of sarcopenia should include assessments of both muscle mass and function, uncertainty remains on the precise cut-off levels applied to determine significant muscle loss and whether distinct criteria should be used for separate diseases, including CKD [12,13]. Thus, the published studies run the risk of potential overreporting or underreporting of the sarcopenia burden in CKD patients, particularly those on chronic dialysis (Table 1).

### 2.1. General Epidemiology

The few available systematic reviews and meta-analyses yield similar results regarding the overall pooled prevalence of sarcopenia in dialysis patients, ranging from 25.6 to 28.5% [7,14,15]. Data from 30 studies published after 2013, including ones defining sarcopenia as the presence of low muscle mass alone, and involving more than 6000 patients undergoing either hemodialysis or peritoneal dialysis, were assessed in the meta-analysis by Shu et al. [7]. Notably, the prevalence of sarcopenia in the studies utilizing the low muscle mass definition was 34.6%, but it dropped to 25.9% when only studies using the combined criteria of low muscle mass and low muscle strength and/or low physical performance were taken into consideration, although the difference did not display statistical significance [7]. These findings align with the results from the meta-analysis by Duarte et al. revealing low muscle mass in 32.2% of the pooled dialysis population [15]. Of note, among the sarcopenia-defining criteria, muscle strength appears to be the most affected trait compared with muscle mass and physical performance, highlighting its significance as a determining factor of sarcopenia in dialysis patients [15]. Accordingly, a significant difference was observed between the dialysis and CKD groups in the meta-analysis by Duarte et al., with one in two hemodialysis patients and one in five CKD patients exhibiting reduced muscle strength [15]. In line with the above, low physical performance was also highly prevalent, affecting more than 45% of dialysis patients [15].

Comparison between the internationally recommended consensus criteria panels indicates that the estimation of the prevalence of sarcopenia as defined by the European Working Group on Sarcopenia in Older People (EWGSOP) might be lower than the prevalence of sarcopenia as defined by the Asian Working Group for Sarcopenia (AWGS) and other criteria [7]. These data agree with results from the meta-analysis by Wathanavasin et al., identifying the highest prevalence of sarcopenia in the AWGS 2019 diagnostic criteria across the different guidelines (36.9%) [14]. In line with the above, a recent analysis of the level of agreement between the revised EWGSOP2 and the Sarcopenia Definitions and Outcomes Consortium (SDOC) in hemodialysis patients from Brazil showed similar prevalence rates of sarcopenia between the consensuses, but with weak agreement, especially within younger patients [16].

### 2.2. Dialysis Modality

Although individual studies indicate that the estimated sarcopenia prevalence rates in hemodialysis patients might be up to two times higher compared with the early CKD stages, a recent meta-analysis by Duarte did not detect any significant differences in the global prevalence of sarcopenia across the CKD stages and dialysis therapies [15]. On the other hand, the prevalence of severe sarcopenia, as defined by the simultaneous presence of low muscle strength, low muscle mass, and low physical performance, displayed a striking difference, affecting 26% of patients undergoing dialysis but only 3% of non-dialysis patients [15].

The available data indicate that peritoneal dialysis confers an advantage on muscle wasting compared with hemodialysis. Accordingly, Wathanavasin et al. reported a pooled sarcopenia prevalence of 26.8% in hemodialysis patients and 17.5% in peritoneal dialysis. Similarly, the meta-analysis by Shu et al. found that sarcopenia was more prevalent in hemodialysis patients than peritoneal dialysis patients, despite the lack of statistical significance [7,14]. Several factors might be implicated in the beneficial impact of peritoneal dialysis on the conservation of muscle mass and muscle function, including the younger patient age; the better health status, with fewer comorbidities and superior cognitive function; as well as the greater preservation of residual kidney function in peritoneal dialysis compared with hemodialysis patients. Yet, the subgroup analyses focusing on dialysis modality in these meta-analyses exhibit limitations due to the small number of PD patients included [7,14].

### 2.3. Impact of Gender

The available studies provide controversial results regarding gender-related differences in the overall prevalence of sarcopenia; however, gender seems to particularly affect specific sarcopenia-related traits such as muscle strength [15]. Accordingly, a retrospective analysis of the results of body composition by DEXA scans from 325 patients receiving peritoneal dialysis showed that the percentage of patients with reduced muscle mass, indexed by dividing by height squared, varied from 2.2–31.3% for female patients and 25.1–75.6% for male patients, depending on the appendicular lean body mass cut-offs recommended by the European and North American clinical guidelines [17]. Using the age- and gender-adjusted NHANES normative data, the prevalence of muscle loss was more pronounced for men than women [17,21]. A study evaluating gender disparities in the prevalence of sarcopenia using the available definitions in 600 hemodialysis patients showed that although male patients had a greater muscle mass compared with female patients, there was a striking decrease in sarcopenia prevalence in women following adjustment for body mass index (BMI) [18]. Notably, relatively more women exhibited reduced hand-grip strength in this study, regardless of the sarcopenia definition utilized and the cut-off criteria applied for gender. These findings support a higher prevalence of muscle weakness compared with muscle mass loss in female hemodialysis patients compared with their male counterparts [18].

### 2.4. Regional and Demographic Differences

Key factors related to sarcopenia, such as body composition as well as factors related to lifestyle, including nutritional patterns and physical activity behavior, vary with ethnicity. Thus, despite the high level of agreement observed between standard anthropometric estimating equations of total body water with bioimpedance analysis measurements, ethnicity seems to affect body composition, including the relative amount of water and adipose tissue content [22,23].

Asian patients are reported to have lower muscle mass compared with the northern European and African American populations, which might confound the estimation of sarcopenia prevalence by the current definitions in these patients [24]. Accordingly, Yoowannakul et al. examined the effect of ethnicity in two separate multiracial cohorts including 600 hemodialysis and 434 peritoneal dialysis patients, respectively [18,19]. Regarding hemodialysis patients, a greater proportion of Asian patients displayed reduced muscle strength compared with both White and Black patients according to the recommended hand-grip strength criteria, which were applicable for all definitions, including the AWGS, EWGS, and FNIH clinical guideline recommendations [18]. Overall, Asian hemodialysis patients displayed a significantly increased prevalence of sarcopenia compared with the other races, even following adjustment of lower muscle mass for a shorter height, as indicated by all three guidelines [18]. Similarly, in the peritoneal dialysis cohort, evaluation by segmental bioimpedance of appendicular lean mass indexed to height showed that Asian women had lower muscle mass adjusted for BMI compared with Black women, whereas Asian men had lower muscle mass compared with both White and Black men [24]. Nonetheless, despite the greater prevalence of reduced appendicular lean mass found in male Asian patients when utilizing the European (>40%) and North American (>35%) criteria, the application of the AWGS cut-offs for the Asian patients markedly reduced the prevalence to 10.5% [24]. In contrast, the meta-analysis by Duarte et al. did not detect any significant differences following analysis stratification by Asian and non-Asian countries or among the European and Asian guidelines; however, it should be noted that both CKD and dialysis patients were included in their data analysis [15]. Finally, Wathanasin et al., in a geographic assessment of the sarcopenia frequency in dialysis patients spanning five macro-regions, Australia, Asia, Europe, and North and South America, showed that the lowest absolute prevalence was observed in North America (15.4%), followed by Australia (17.9%), South America (20.4%), Asia (27.9%), and Europe (29.1%) [14]. Therefore, the conclusion may be drawn that the currently available definitions of sarcopenia cannot be uniformly applied to racially diverse populations, and the development of updated definition panels should take into consideration ethnicity-matched anthropometric normative data. Of note, there is a paucity of data from the African continent, and future studies are required to shed light on the epidemiological burden and the specific features of sarcopenia in African dialysis patients [25].

## 3. Risk Factors and Pathogenesis of Sarcopenia in Dialysis Patients

**Figure 1:**
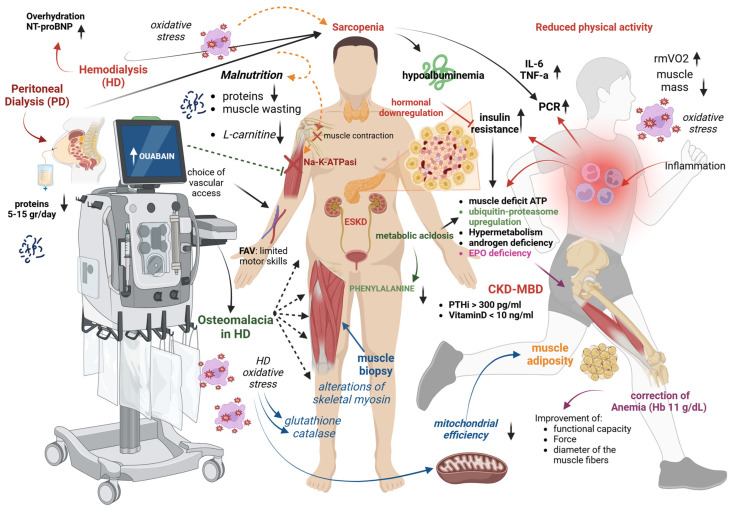
Multifactorial pathogenesis of sarcopenia in dialysis patients. This figure illustrates the multifactorial mechanisms contributing to sarcopenia in dialysis patients. It highlights the interplay between hemodialysis (HD) and peritoneal dialysis (PD) with factors such as malnutrition, oxidative stress, metabolic acidosis, and hormonal dysregulation. The diagram also emphasizes the role of chronic kidney disease–mineral and bone disorder (CKD-MBD), inflammation, and mitochondrial dysfunction in muscle wasting and reduced physical activity. Key elements include insulin resistance, ubiquitin–proteasome system upregulation, and erythropoietin (EPO) deficiency, all of which contribute to impaired muscle function and mass loss.

Sarcopenia in dialysis patients has a complex and multifaceted origin, involving general and dialysis-specific factors such as chronic inflammation, oxidative stress, metabolic acidosis, and hormonal imbalances. Additionally, uremia-induced protein-energy wasting and the physical and psychological burden of dialysis therapy further accelerate muscle loss. These mechanisms are compounded by age-related changes, comorbidities, and reduced physical activity, creating a multifactorial syndrome that characterizes sarcopenia in the ESKD-dialysis setting (Figure 1).

### 3.1. Reduced Physical Activity

Poor physical activity is largely prevalent in dialysis patients and is related to worse outcomes and mortality. The main causes include fatigue, muscle weakness, chronic inflammation, and the physical toll of their underlying kidney disease [26]. Regular hemodialysis sessions can lead to a sedentary lifestyle, as patients spend significant time receiving treatment and recovering afterward [27]. Beyond physical factors, psychological barriers play an equally important role. Depression and anxiety are highly prevalent in this population, stemming from the chronic nature of kidney disease, the burden of frequent dialysis sessions, and uncertainty about their prognosis. These mental health challenges can lead to apathy, low motivation, and a sense of helplessness, making it difficult for patients to engage in physical activity [28]. Fear of injury or worsening their health may also discourage exercise. Furthermore, social isolation and a perceived stigma related to their illness can reduce their willingness to participate in group or community activities. These psychological barriers contribute to a sedentary lifestyle, which exacerbates muscle wasting and increases the risk of sarcopenia, creating a cycle of physical and emotional decline.

Low physical activity may trigger deconditioning, defined as a decline in physical function and a progressive loss of muscle mass due to physical inactivity. Resting muscle oxygen consumption (rmVO^2^), which determines the muscle’s ability to consume oxygen from the blood, might serve as a non-invasive marker quantifying both the degree and the potential reversibility of myopathy in dialysis [29]. Compared with healthy controls, dialysis patients have a two-fold increase in rmVO^2^; physical exercise in dialysis causes a 23% reduction in rmVO^2^ values, suggesting that exercise might ameliorate muscle dysfunction in HD.

### 3.2. Inflammation

The strong link between inflammation and dialysis-related sarcopenia has been repeatedly reported, with C-reactive protein among the inflammatory markers emerging as the most common predictor of sarcopenia in these patients [30,31]. Inflammation is a common feature in advanced CKD and is more exacerbated in ESKD. In this stage, there is an increase in the circulating levels of catabolic inflammatory cytokines, such as C-reactive protein (CRP), interleukin-6 (IL-6), and tumor necrosis factor-alpha (TNF-α), which trigger catabolic processes, including the downregulation of albumin synthesis, protein degradation, and the promotion of anorexia [32,33]. However, even in well-dialyzed patients with preserved appetite, these pro-inflammatory cytokines might also shift the protein generation from muscle to acute-phase proteins, leading to overall hypoalbuminemia, loss of body weight, and muscle deficit, possibly through upregulation of the ATP–ubiquitin–proteasome molecular pathway, hypermetabolism, and insulin resistance [34]. Therefore, inflammation represents a major causal factor linking malnutrition and protein-energy wasting to muscle dysfunction and cardiovascular disease (CVD) in dialysis patients. A multicenter, cross-sectional study including 209 HD patients showed that sarcopenia was closely associated with increased high-sensitive CRP levels [35].

### 3.3. Fluid Overload and Dialysis Prescription

Fluid overload has been associated with muscle mass decline and sarcopenia development in dialysis patients [36,37]. Recent findings indicate that elevated N-terminal pro-B-type natriuretic peptide (NT-proBNP), a marker of overhydration, might serve as an independent predictor for lean body mass decrease in hemodialysis patients [38,39]. These findings are in agreement with results from the meta-analysis by Wathanasin et al., which revealed a time-dependent effect with regard to the dialysis procedure in the diagnosis of sarcopenia (21.5% before hemodialysis to 27.8% post hemodialysis) [14]. Indeed, body composition evaluation before the hemodialysis session might generate less-reliable results and overestimate the muscle mass due to increased water content [40]. Even though data regarding the influence of convective treatments on sarcopenia are limited, a recent study showed that patients undergoing high-volume hemodiafiltration displayed larger muscle mass compared with those undergoing high-flux hemodialysis [41].

### 3.4. Malnutrition and Protein Loss

Malnutrition is common in dialysis patients due to reduced appetite, dietary restrictions, and the metabolic effects of kidney failure. It can lead to muscle wasting, weakened immunity, and poorer treatment outcomes. On top of this, dialysis treatment may be associated with increased protein waste during the procedure. Daily peritoneal protein losses in peritoneal dialysis patients vary between 5 and 15 g daily, whereas each hemodialysis session removes 6 to 12 g of amino acids; however, the clinical consequences remain ambiguous [42,43]. Even though most studies show an inverse correlation between serum albumin levels and sarcopenia prevalence, no association has been observed between protein losses during the dialysis procedure and muscle mass or strength [44].

### 3.5. L-Carnitine Deficit

Hemodialysis patients with sarcopenia have lower levels of carnitine, an essential co-factor for the oxidation process of long-chain fatty acids and energy generation in the skeletal muscles, compared with those without, thus supporting a possible role for carnitine as a biomarker for sarcopenia assessment in this setting [45]. Additionally, L-carnitine plays a crucial role in mitochondrial function and energy metabolism and may influence muscle protein synthesis pathways, particularly through the regulation of the mTOR signaling pathway. By modulating mitochondrial efficiency and reducing oxidative stress, L-carnitine could contribute to maintaining muscle mass in dialysis patients [46]. Supplementation of L-carnitine in HD increased muscle strength, as assessed by rating scales and questionnaires, but it had no effect on endurance [47] or muscle levels of long-chain fatty acids oxidation (FAO). However, L-carnitine failed to show any beneficial effects when muscle function and bioenergetics were assessed by magnetic resonance imaging and near-infrared spectroscopy [48].

### 3.6. Altered Mineral Metabolism

A significant body of experimental and clinical evidence from the general population indicates the key role of parathormone (PTH) and vitamin D on skeletal muscle health; thus, CKD-MBD components have emerged as important sarcopenia risk factors in dialysis patients [49,50]. A linear association between elevated serum levels of intact PTH and low appendicular skeletal muscle index was found in a cohort of peritoneal dialysis patients, with individuals with intact PTH levels above 300 pg/mL displaying a 4.74-fold increased odds ratio (OR) (95% CI: 1.53–14.70) for sarcopenia compared with those having intact PTH levels less than 150 pg/mL [36]. On the other hand, considering that Wathanasin et al. found lower serum PTH levels in dialysis patients with sarcopenia, the existence of a U-shaped relationship between PTH and sarcopenia should be taken into consideration [14]. Likewise, an independent association was shown between lower serum 25 (OH) vitamin D levels with muscle mass and strength impairment in both peritoneal dialysis and hemodialysis patients, with serum 25 (OH) vitamin D levels lower than 10 ng/mL displaying an OR of 5.60 (95% CI: 1.52–20.57) for sarcopenia [51,52]. Risk prediction models incorporating various risk factors have been developed in dialysis patients to identify those at higher risk for sarcopenia development; still, future studies are required to validate their performance [53]. ESKD is a state of vitamin D deficiency and impaired metabolism of calcium, which is essential for the upregulation of myofibrillar ATPase activity and the skeletal muscle excitation–contraction coupling. Depletion of vitamin D has been linked with muscle weakness in various settings, including vitamin D-induced rickets and statin-associated myopathy [54,55]. In HD, advanced osteomalacia promotes muscle fiber atrophy and severe proximal myopathy [56], whereas 25 (OH) D levels are associated with lower extremity muscle force [57]. Treatment with native vitamin D or synthetic analogs prevents fractures, reduces falls, and in HD patients, improves myopathy symptoms [58]. Notably, intoxication with aluminum (which was first used as a phosphate binder) commonly causes proximal muscle weakness and bone pain; however, the affected patients are usually asymptomatic at first. Both primary [59] and secondary hyperparathyroidism (SHP) [60] have been associated with neuropathy, muscle loss, and atrophy; however, only the myopathy attributed to primary hyperparathyroidism is reversible after suppression of PTH, which implies causality [61]. Yet, in dialysis, SHP and vitamin D deficiency are interrelated entities that are difficult to distinguish.

### 3.7. Uremic Metabolic Alterations

Uremic middle molecular weight compounds are not effectively removed by standard HD and might be toxic to skeletal muscles [62,63,64]. This was first shown in 1969, when induced uremic intoxication in animals caused muscle weakness and limited physical activity [65]. The majority of ESKD patients present increased levels of ouabain, an endogenous factor similar to digitoxin, which inhibits the sodium–potassium ion pump (Na-K-ATPase), causing intracellular changes in electrolytes that directly affect muscle contractility [66,67]. Moreover, the uremic environment might affect the FAO, which represents the main energy source of skeletal muscle energy, thus leading to uremic myopathy and sarcopenia [62].

### 3.8. Metabolic Acidosis

Metabolic acidosis (MA) resulting from the overproduction of nonvolatile acids, high loss of bicarbonate, and reduced renal removal of acid, is a common feature in advanced CKD. Oral correction of MA, in addition to the preservation of kidney function, might also improve muscle mass [68,69]. In ESKD, MA occurs in the pattern of low to moderate intermittent state and promotes muscle protein breakdown and muscle atrophy through overactivation of the ATP–ubiquitin–proteasome molecular pathway [70], increased endogenous generation of glucocorticoids, and suppression of the insulin anabolic effects [71]. Despite the use of bicarbonate dialysate in HD patients with adequate Kt/v, pre-dialysis circulating carbon dioxide was inversely associated with protein catabolic rate [72]. A crossover study in 16 HD patients showed that correction of MA reduced muscle net phenylalanine efflux, thus suggesting that MA increases protein degradation in skeletal muscle [73].

### 3.9. Oxidative Stress

Dialysis is a state of increased oxidative stress (OS) due to the overproduction of reactive oxygen species (ROS) triggered by the contact of the patient’s blood with the bioincompatible dialysate and filter [74]. Other factors promoting OS include intravenous iron and erythropoietin (EPO) agents, the use of central venous catheters, reduced dietary intake of antioxidants, and the loss of antioxidant trace elements and vitamins through the dialysis filter. Dialysis-induced OS triggers a muscle catabolic state, suppresses muscle production, reduces muscle contractility, and downregulates sarcomeric protein expression [75,76]. These detrimental effects might be either acute or long-term and are usually irreversible. Acutely, ROS and elevated nitrogen species that are generated during an HD session decrease Ca^2+^ sensitivity and cause oxidative modification of the sarcoplasmic reticulum Ca^2+^ channels [77,78], thus leading to muscle fatigue; conversely, long-term exposure to free radicals causes a progressive oxidative modification of the structure and function of biomolecules (lipids, proteins, genes, carbohydrates) that are essential for muscle contractility [78]. Experimental uremia triggers critical alterations in skeletal myosin heavy chains [79], which is confirmed by clinical data; a skeletal muscle biopsy study showed increased oxidative injury of proteins and lipids (assessed by protein carbonyls and malondialdehyde) [80] and several deletions of mitochondrial DNA mutations in the muscles of dialysis patients that were attributed to accelerated protein degradation and elevated circulating inflammatory cytokines. It is suggested that the muscle of HD patients undergoes adaptive changes in the content and activity of glutathione, catalase, and heat shock protein, which might be triggered by long-term OS [81]. These findings suggest a pivotal role of OS in the pathogenesis of uremic skeletal myopathy [75].

### 3.10. Mitochondrial Dysfunction

Mitochondria are organelles playing a pivotal role in muscle homeostasis. The muscle mitochondria of HD patients have decreased content and DNA copies, altered structures with excessive fragmentation, and impaired dynamics and biogenesis capacity, factors leading to functional decline [82]. These alterations in mitochondrial metabolism and function, irrespective of oxygen supply, lead to sarcopenia. In HD, OS, along with inflammation, reduces mitochondrial efficiency and results in mitochondrial uncoupling, characterized by decreased coupling of the adenosine triphosphate generated with oxygen consumed [83,84,85]. These alterations might occur before the decline in physical function and correlate with decreased physical performance and increased intramuscular adiposity [86].

### 3.11. Endocrine Alterations

Accelerated protein catabolism might be promoted by the downregulation of anabolic hormones, including insulin. Insulin resistance occurs in ESKD due to inflammation, central adiposity, and other metabolic changes. In both diabetics and non-diabetics, as well as non-obese HD patients, insulin resistance is a major determinant of skeletal muscle protein turnover and increased protein degradation, irrespective of inflammation status [87]. All HD patients (irrespective of gender) have androgen deficiency, which promotes myopathy. Before the EPO era, anabolic steroids were used for the treatment of renal anemia, and they have been reported to increase muscle mass and strength and improve physical strength and the parameters of body composition [88,89]. A large longitudinal study enrolling 440 HD patients from 14 centers reported that a 50% reduced free testosterone concentration was associated with a 1.40-fold higher risk for frailty (defined by grip strength and gait speed) and a 1.55-fold risk of sarcopenia [90]. A 2 × 2 randomized controlled trial (RCT) of resistance exercise training and supplementation of the anabolic steroid nandrolone decanoate in 79 stable HD patients showed that nandrolone increased lean body mass by 3.1 kg and the muscle mass of the quadriceps. In contrast, exercise increased only muscle size [91].

### 3.12. Other Factors

A deficiency of EPO might contribute to muscle impairment either because EPO affects the muscles directly or because the resulting renal anemia reduces muscle oxygen delivery. Correction of anemia in HD patients improves endurance, functional ability, and leg physical strength measured both isokinetically and isometrically [92]. Treatment with EPO improves the diameter of muscle fibers and repairs muscle structure abnormalities [93], and when hemoglobin values of 11 g/dL are achieved, there is a 10–30% improvement in leg and arm muscle strength [94].

The choice of vascular access might affect muscle function locally. An arteriovenous fistula in the forearm will most likely limit both gross and fine motor skills of this arm. Moreover, restrictions regarding heavy lifting might lead to a decrease in muscle mass and reduce patient activity.

## 4. Clinical Consequences of Sarcopenia in Dialysis Patients

The progressive and debilitating loss of skeletal muscle mass and strength has major effects on short- and long-term comorbidities among dialysis patients, including physical impairment and profound impacts on health, quality of life, cardiovascular diseases, and overall survival.

### 4.1. Physical Complications

The physical complications of sarcopenia are immediately notable in dialysis patients and include decreased mobility, decreased exercise capacity, and challenges in carrying out activities of daily living (ADLs). Muscle wasting impairs strength and endurance, making daily tasks such as walking, climbing stairs, and maintaining balance harder. As for the elderly population, the decrease in muscle strength occurs rapidly compared with the muscle mass loss among CKD patients; in addition, alterations in muscle quality with muscle fiber atrophy were described in an animal model of progressive kidney disease [95]. Similar findings were reported by Fahal et al., who analyzed the prevalence and causes of muscle weakness in dialysis patients by examining the quadriceps muscle force and contractile properties, reporting interesting results. Quadriceps muscle strength was lower in hemodialysis patients compared with healthy subjects, especially in those who were malnourished, and their muscle mass exhibited a slower rate of relaxation; moreover, muscle biopsies revealed important changes in muscle quality, and they described a significant atrophy in type I and II fibers in around 40% of the study population [96]. Consistent with these results, Johansen et al. investigated muscle atrophy among hemodialysis patients and highlighted a significant atrophy and an increase in non-contractile tissue in the muscle mass of such populations, suggesting that muscle function loss is more correlated with muscle quality impairment than a progressive muscle loss [97]. In addition, several longitudinal studies indicate that loss of muscle strength represents a strong predictor of several complications among patients on chronic dialysis, including the risk of hospitalization and mortality [15,98].

### 4.2. Reduced Quality of Life

The decline in physical function in sarcopenic patients translates into a markedly reduced quality of life. Health-related quality of life (HRQoL) assessments consistently show lower scores in hemodialysis patients with sarcopenia, with physical, emotional, and social consequences. The loss of independence and the need for caregivers for daily needs affect patients’ sense of dignity and self-esteem; additionally, the chronic stress related to the hemodialysis condition, associated with the frustration from physical limitations, often leads to depression and anxiety, compounding the psychological burden of living with ESKD. In a clinical study including 113 patients on chronic hemodialysis, lower appendicular lean mass (ALM) measured by segmental bioimpedance correlated with higher depression, anxiety, and self-reported general health scores (Beck Depression Inventory-II, BDI-II; Hospital Anxiety and Depression Scale, HADS) [99].

### 4.3. Frailty and Risk of Falls

The loss of muscle strength and coordination significantly compromises mobility in dialysis patients. The reduced muscle mass and strength contribute to the development of frailty, a clinical syndrome characterized by increased vulnerability related to the loss of specific features or functions [100]. Frail patients are more likely to carry an increased risk for poor health outcomes including falls, infections, incident disability, hospitalization, complications from surgical procedures, and overall mortality. Frailty was evaluated in a cross-sectional and longitudinal study on 119 patients on peritoneal dialysis using the Clinical Frailty Scale (CFS). Patients with frailty exhibited significantly lower skeletal muscle mass index (SMI), hand-grip strength, and usual walking speed [101]; in addition, in a multivariate logistic regression model, sarcopenia strongly correlated with CFS after adjusting for age, gender, BMI, and normalized protein equivalent nitrogen appearance (nPNA) (OR 12.2, 95% CI: 2.27–65.5, *p* = 0.003) [101].

Sarcopenic patients are at an increased risk of falls with a consequent high likelihood of fractures and related complications in this population; repeated falls and injuries further reduce mobility, increasing dependence and reducing quality of life. In this scenario, dialysis patients with sarcopenia presented weakened musculature, poor balance, and impaired coordination, leading to an increased risk of fracture. Falls often result in fractures, particularly of the hip and pelvis, which are associated with high morbidity and prolonged recovery times. In a cross-sectional study enrolling 100 hemodialysis patients in Buenos Aires, sarcopenia was significant among the study population (43.7% of patients), and those with lower hand-grip strength (NGS) were characterized by a high prevalence of falls (2 or more falls in 40% of patients) [102]. Additionally, hospitalization for fall-related injuries can complicate disease management, due to the need to adapt the dialysis schedule, and further exacerbate sarcopenia through inactivity and bed rest.

Another critical point is the impact of sarcopenia and reduced mobility in limiting participation in social activities, with consequent isolation and a decline in mental health. In a cross-sectional study including 238 patients on chronic hemodialysis, the degree of dependency in activities of daily living (ADLs) was assessed and associated with the prevalence of sarcopenia in that population [103]; severe sarcopenia (30.7% of the study population) was closely associated with dependency in basic ADLs (OR 4.68, 95% CI: 2.11–10.40) and instrumental ADLs (OR 3.24, 95% CI: 1.61–6.53), and gait speed was the predominant component affecting dependency in ADLs [103].

For many patients, the loss of autonomy is one of the most devastating consequences of sarcopenia, underscoring the need for targeted interventions designed to improve muscle mass and to reduce levels of depression and anxiety and improve the general quality of life, preserving physical function and social engagement.

### 4.4. Increased Hospitalization Rate

Sarcopenia significantly increases the risk of hospitalization in dialysis patients, with wide-ranging consequences for health and healthcare utilization.

Few studies have reported on the impact of sarcopenia on the risk of hospitalization among hemodialysis patients [104,105]. In a 3-year longitudinal study including 126 chronic hemodialysis patients, although severe sarcopenia was not correlated with hospitalization, a lower hand-grip strength (HGS) and slow gait speed were significantly associated with an increased risk of hospitalization [105]. Conversely, the risk of hospitalization was reported as significantly higher in hemodialysis patients with sarcopenia in a longitudinal study of 170 patients from 6 dialysis centers in Brazil [104].

Sarcopenia demonstrates a close association with PEW, a clinical condition marked by severe malnutrition and consequent immune dysfunction [106,107]. This immunosuppression represents a critical issue in dialysis patients, since the increased vulnerability to infections may increase the risk of peritonitis in individuals on peritoneal dialysis and vascular access-related infections in hemodialysis patients. Furthermore, the reduced recovery potential in frail and sarcopenic patients is associated with impaired wound healing and a high risk for post-surgical complications, and it substantially raises the probability of hospital readmissions.

The cumulative effects of malnutrition, immune dysfunction with infectious complications, physical injuries, disability, and delayed wound resolution extend hospitalization length of stay for dialysis patients with sarcopenia. In this scenario, a deleterious vicious cycle occurs, as the prolonged hospital stays exacerbate muscle atrophy through disuse and suboptimal nutritional support, worsening sarcopenia. In this scenario, an integrative and multidisciplinary approach is required, including nutritional rehabilitation and structured physical therapy for hospitalized patients to disrupt this vicious cycle.

### 4.5. Mortality and Cardiovascular Risk

The link between sarcopenia and mortality risk in dialysis patients is well reported, with extensive evidence suggesting its role as an independent determinant of adverse outcomes. Notably, cardiovascular disease, the predominant mortality cause among hemodialysis patients, shares significant overlaps with sarcopenia. In a cohort study including 308 patients, the presence of diabetes and sarcopenia correlated with the all-cause mortality rate, especially in the older population (>60 years) [108]. In addition, although muscle mass and strength are prevalent in the dialysis population, they are often not congruent, as loss of muscle strength may occur even in the context of normal muscle mass; patients with low muscle strength were at an increased risk of mortality, irrespective of their muscle mass [10,14].

A recent systematic review and meta-analysis including 41 studies with 7576 dialysis patients analyzed the impact of sarcopenia on cardiovascular events and overall mortality [14]; sarcopenia evaluated by both criteria (low muscle mass, LMM; low muscle strength, LMS) was significantly associated with an increased risk for mortality (OR 1.83, 95% CI: 1.44 to 2.39, *p* < 0.001).

Two milestone studies investigated the relationship between sarcopenia and cardiovascular events [109,110], both reporting a strong association. Conversely, Baltaci et al. analyzed the impact of sarcopenia on pre-atherosclerotic markers, cardiovascular events, and mortality among 106 hemodialysis patients [108]; no differences in the carotid intima media thickness and pulse wave velocity measures were reported between patients with and without sarcopenia, and no differences in cardiovascular events and overall deaths were highlighted during the 2-year follow-up period, except for a significant increase in the mortality risk among sarcopenic PD patients [108].

Chronic systemic inflammation, characterized by elevated pro-inflammatory cytokines such as interleukin-6 (IL-6) and tumor necrosis factor-alpha (TNF-α), serves as a pivotal intermediary between sarcopenia and cardiovascular complications [98]. These inflammatory markers induce endothelial dysfunction, arterial rigidity, and accelerated atherogenesis, collectively amplifying cardiovascular event risk. Concurrently, inflammation-driven muscle degradation perpetuates the interplay between sarcopenia and systemic cardiovascular compromise.

In addition, PEW synergistically intensifies cardiovascular risk by deranging metabolic equilibrium [106]. The resultant malnutrition and muscle catabolism impair glucose homeostasis, fostering insulin resistance and increasing susceptibility to type 2 diabetes mellitus. These metabolic aberrations accelerate the trajectory of cardiovascular pathology, thereby magnifying mortality and cardiovascular risks among sarcopenic dialysis patients.

Table 2 summarizes findings from the main clinical sarcopenia studies in dialysis patients.

## 5. Methods for Assessing Sarcopenia in Dialysis Patients

**Figure 2:**
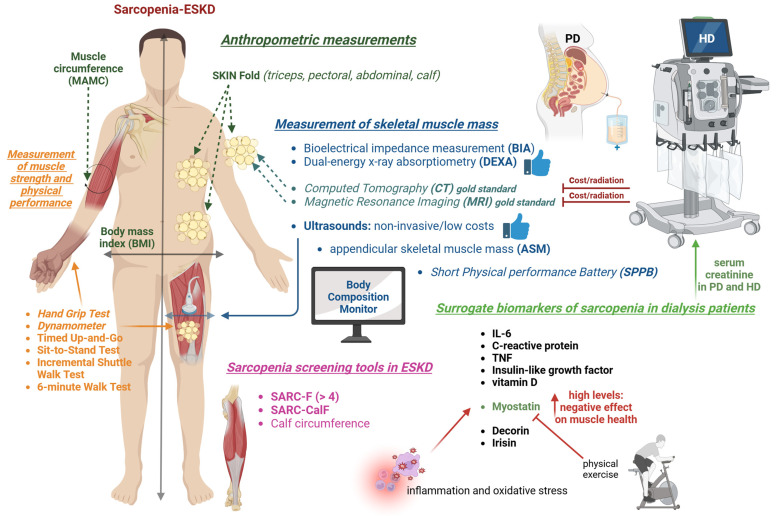
Main methods for assessing sarcopenia in dialysis patients. This figure presents an overview of methods used to assess sarcopenia in dialysis patients, categorized into anthropometric measurements (muscle circumference, BMI, skinfold thickness), skeletal muscle mass evaluation (bioelectrical impedance analysis, DEXA, CT, MRI, and ultrasound), functional performance tests (hand-grip strength, dynamometer, timed up-and-go, sit-to-stand test, incremental shuttle walk test, and six-minute walk test). Surrogate biomarkers associated with sarcopenia, including inflammatory markers (IL-6, TNF, C-reactive protein), myostatin, and insulin-like growth factor are also highlighted.

The appropriate assessment of sarcopenia in dialysis patients is crucial for early identification and management. Numerous methods exist to evaluate sarcopenia, each with distinct advantages and limitations. These methods broadly encompass imaging techniques, anthropometric and physical performance tests, and biochemical markers, reflecting the multifaceted nature of sarcopenia (Figure 2). The choice of assessment method often depends on clinical context, resource availability, and patient-specific factors.

### 5.1. Anthropometric Measurements

Skinfold thickness measurements are notable for their simplicity and non-invasive nature and could be useful for screening. These measurements involve assessing subcutaneous fat at specific sites such as the triceps (particularly informative in women), the pectoral region (commonly measured in men), the abdominal or supra-iliac area, the thigh, and additional sites such as the biceps, subscapular region, and calf. Among these, the mid-arm muscle circumference (MAMC) is a simple, straightforward, and highly clinically feasible method for assessing skeletal muscle mass by measuring the mid-arm circumference and triceps skinfold thickness at the midpoint between the acromion and olecranon. The MAMC is calculated using the following formula: mid-arm circumference (cm) − π × triceps skinfold (cm). However, this method is susceptible to measurement bias and interrater variability and is insensitive to detect subtle muscle mass or quality changes. In general, all skinfold assessments may have limited accuracy in dialysis patients with significant fluid retention, as excess extracellular fluid can distort the real thickness of subcutaneous fat layers.

Body mass index (BMI) is another frequently utilized measure in sarcopenia assessment, but it cannot differentiate between lean mass, fat mass, and fluid compartments, which may be problematic in dialysis patients. A high BMI in these subjects might obscure significant sarcopenia if the excess weight is predominantly fat or fluid, while a low BMI might reflect severe muscle wasting but could also result from malnutrition or other comorbidities. Body fat percentage, derived from skinfold measurements, offers additional information. However, like other measures, it must be interpreted cautiously. Excess fat mass, often observed in dialysis patients due to altered metabolism, may mask significant losses in lean mass, potentially leading to an underestimation of sarcopenia severity.

### 5.2. Measurement of Skeletal Muscle Mass

A more accurate, instrumental assessment of skeletal muscle mass is crucial for sarcopenia confirmation. Various tools are available, including bioelectrical impedance analysis (BIA), dual-energy X-ray absorptiometry (DEXA), computed tomography (CT), magnetic resonance imaging (MRI), and ultrasound, each with unique advantages and limitations [136].

CT and MRI are the ideal and gold-standard tools for sarcopenia assessment, with high accuracy and sensitivity. They quantify muscle mass by analyzing the cross-sectional area and evaluate muscle quality by detecting intramuscular fat infiltration [137]. However, their widespread application and use for repeated measurements in longitudinal follow-ups are constrained due to the high cost of both image modalities and the significant radiation exposure associated with CT.

BIA and DEXA are two widely recommended methods for measuring skeletal muscle mass, as endorsed by current sarcopenia consensus guidelines [138,139,140,141]. DEXA measures skeletal muscle mass by using low-dose X-ray beams at two energy levels to differentiate bone, lean tissue, and fat tissue, whereas BIA estimates skeletal muscle mass based on the electrical properties of body tissues by delivering harmless electrical current. However, in patients with advanced CKD and ESKD, hydration status significantly affects the accuracy of skeletal muscle measurements, often leading to overestimation. This impact may be minimized by performing measurements after hemodialysis sessions or with an empty abdomen in peritoneal dialysis patients. Furthermore, the whole-body multi-frequency bioimpedance spectroscopy devices, such as Body Composition Monitor (Fresenius Medical Care, Bad Homburg, Germany), are increasingly used to evaluate body composition in dialysis patients, as they differentiate between intracellular and extracellular fluid and offer a more precise assessment of hydration status [142,143]. In this regard, skeletal muscle mass measurements are less influenced by hydration status. Beyond lean tissue mass measurement, a formula has been developed to estimate appendicular skeletal muscle mass (ASM) based on total body water, age, gender, and body weight [124]. This aligns with the EWGSOP2 and 2019 AWGS criteria, which prioritize the assessment of ASM over total skeletal muscle mass [139,140].

Nevertheless, the question of whether multi-frequency bioimpedance devices are superior to single-frequency devices in accurately measuring skeletal muscle mass in ESKD patients remains inconclusive. An observational study in PD patients demonstrated that using DEXA as the reference standard, a single-frequency bioimpedance device might outperform whole-body multi-frequency bioimpedance spectroscopy in assessing fat-free mass and fat mass [144]. For this reason, it is currently impossible to recommend specific BIA devices for ESKD patients. Given the poor agreement between single-frequency and multi-frequency bioimpedance devices, these devices should not be used interchangeably [145]. For longitudinal body composition monitoring in ESKD patients, it is advisable to use the same device to minimize measurement variability.

Longitudinal assessments of body composition change are critical for the early detection of muscle wasting, enabling timely interventions to prevent its progression. This is particularly important in ESKD patients, who often exhibit anabolic resistance, making it challenging to reverse sarcopenia effectively once it has developed. Moreover, longitudinal monitoring provides more prognostic information than cross-sectional analyses. A 6-year longitudinal observational study involving 160 PD patients revealed that lean tissue loss or fat tissue gain significantly increased mortality risk, while lean mass gain with minimal fat tissue accumulation was associated with the lowest mortality risk [146]. Notably, most patients experiencing lean tissue loss maintained stable body weight and body mass index due to compensatory fat tissue gain. This finding underscores the limitations of weight and body mass index as indicators of muscle changes, emphasizing the indispensable role of direct body composition measurements.

Ultrasound has emerged as a promising tool for sarcopenia evaluation in recent years due to its non-invasive nature, timely results, affordability, and ability to assess muscle quantity and quality, making it suitable for clinical and research applications [147]. Muscle quantity can be measured through muscle thickness and the cross-sectional area of the lower extremities, commonly quadriceps muscles, while muscle quality can be evaluated via echogenicity [148]. A hyperechoic appearance indicates intramuscular fat infiltration and reduced muscle quality. This versatile modality holds great potential for facilitating sarcopenia assessment. Further research is needed to validate standardized protocols and establish the cut-offs of parameters.

In HD patients, ultrasound-derived muscle mass strongly correlates with BIA-derived measurements. It is independently associated with hand-grip strength and physical performance metrics, including gait speed, chair stand time, and Short Physical Performance Battery (SPPB) scores. Among patients diagnosed with sarcopenia based on ultrasound criteria, 96% also met the BIA-based diagnostic criteria [149]. Additionally, the thickness of the vastus intermedius muscle has been linked to mortality risk in HD patients [150,151]. This finding supports the validity and feasibility of ultrasound for assessing muscle quantity in dialysis populations.

### 5.3. Measurement of Muscle Strength and Physical Performance

Muscle strength is typically assessed using hand-grip or lower extremity strength with a dynamometer, while usual gait speed over 4 or 6 m is commonly used to evaluate physical performance. Additional methods such as the SPPB, timed up-and-go test, repeated sit-to-stand tests, incremental shuttle walk test, and the six-minute walk test are also frequently employed to assess physical performance in patients with CKD and ESKD [152]. For HD patients, these assessments are preferably conducted before dialysis, as HD sessions may negatively impact muscle strength and physical performance [153].

ESKD patients universally exhibit lower muscle strength and poorer physical performance than normal subjects [154]. Thus, it is not surprising that the diagnosis of sarcopenia in ESKD patients is driven by low skeletal muscle mass [155]. The diagnosis of sarcopenia may overlook ESKD patients with isolated muscle weakness and poor physical performance, who exhibit high mortality risks despite normal muscle mass. In ESKD patients undergoing dialysis, those with normal muscle mass but reduced muscle strength and slow gait speed independently predicted poor outcomes [10,105,119]. Therefore, in addition to addressing patients suffering from sarcopenia, those with normal muscle mass but reduced muscle strength or poor physical performance, classified as “sarcopenia probable” by EWGSOP2 and “possible sarcopenia” in the 2019 AWGS, also represent a critical high-risk group. Identifying these patients and providing timely and appropriate interventions is crucial for improving their long-term prognosis.

In addition, adjusting the cut-offs for muscle strength and physical performance for ESKD patients is prudent given the unique characteristics of uremic myopathy that differentiate them from the general geriatric population. For example, in a Chinese PD cohort, optimal hand-grip strength cut-offs were identified as 24.5 kg for men and 14 kg for women based on their predictive value for mortality risk [156]. Similarly, a Brazilian dialysis cohort determined hand-grip strength cut-offs predictive of mortality as 22.5 kg for men and 7 kg for women [157]. Furthermore, in a Belgian HD cohort, a 6-min walk test distance of less than 298 m was established as a cut-off associated with increased mortality risk [158]. A more recent study established reference values for muscle strength and physical performance through hand-grip strength, sit-to-stand tests, and walking speed in a large HD cohort [159]. Men generally outperformed women in strength and speed, though younger men (18–49 years) showed little age-related decline. Older patients of both sexes exhibited poorer performance, and those with arteriovenous fistulas had weaker grip strength in the affected arm. Despite being interesting, these findings necessitate external validation in other cohorts to confirm their widespread applicability and prognostic usefulness in the dialysis setting.

### 5.4. Sarcopenia Screening Tools

Three screening tools for sarcopenia—SARC-F, SARC-CalF, and calf circumference—recommended by the AWGS 2019 [140] have been applied to dialysis populations. The SARC-F questionnaire evaluates five components: strength, walking assistance, chair rising, stair climbing, and falls. Each item is scored from 0 (no difficulty) to 2 (a lot or unable), with a total score of 10. A score of ≥4 indicates a high risk of sarcopenia [160]. In prevalent HD patients, we demonstrated that higher SARC-F scores are significantly associated with increased mortality [161]. However, a cut-off score of ≥4 has limited sensitivity for detecting mild to moderate sarcopenia. Lowering the threshold to ≥1 enhances sensitivity, enabling earlier detection of sarcopenia in ESKD patients. To enhance the diagnostic performance of SARC-F, the SARC-CalF tool integrates calf circumference measurement. A score of 10 is added to the original SARC-F total score if the calf circumference is <34 cm for men or <33 cm for women. This modification improves the tool’s ability to identify sarcopenia [162]. In a recent study of PD patients, calf circumference emerged as the most reliable predictor of sarcopenia, outperforming both SARC-F and SARC-CalF [163]. This finding is consistent with results from another study in Japanese HD patients [164], further reinforcing calf circumference as a simple and effective screening tool.

### 5.5. Surrogate Biomarkers of Sarcopenia

Other biomarkers for sarcopenia encompass a diverse range of biological processes. These include inflammatory markers such as interleukin-6, C-reactive protein, and tumor necrosis factor-α; hormones such as insulin-like growth factor-1, vitamin D, and testosterone; antioxidants such as carotenoids and α-tocopherol; and myokines, such as myostatin, decorin, and irisin [165,166]. However, their utility as diagnostic markers for sarcopenia remains uncertain. Among these, serum myostatin shows promise. Myostatin, a key regulatory myokine for muscle health, is activated by inflammation, uremic toxins, and oxidative stress but suppressed by physical exercise [167]. It contributes to muscle wasting, metabolic disturbances, insulin resistance, and the progression of atherosclerosis. Based on these roles, it is reasonable to hypothesize that elevated serum myostatin levels would negatively impact muscle health. Interestingly, accumulated observational studies in CKD, HD, and PD populations have consistently demonstrated that serum myostatin levels are positively associated with skeletal muscle mass and hand-grip strength [167]. This finding is further supported by an intervention study showing increased serum myostatin levels after 12 months of intradialytic exercise training [168] and evidence that lower serum myostatin levels at dialysis initiation predict higher 1-year hospitalization and mortality rates [169]. In this regard, serum myostatin can be recognized as a surrogate marker for muscle mass and strength. Unfortunately, the correlation coefficients between myostatin and skeletal muscle were weak to moderate and insufficient for precise skeletal muscle mass prediction. Some studies have explored using multiple biomarkers in combination to enhance the predictive accuracy of sarcopenia in non-CKD populations [170,171]. In a case-control study, using a combination of four serum biomarkers—interleukin-6, secreted protein acidic and rich in cysteine, macrophage migration inhibitory factor, and insulin-like growth factor 1—enhanced the accuracy of sarcopenia diagnosis. These biomarkers were selected after screening 21 potential candidates [171]. Another study demonstrated that a panel of six novel biomarkers—irisin, pro-peptide of type III procollagen, c-terminal agrin fragment-22, osteonectin, fatty acid-binding protein-3, and macrophage migration inhibitory factor—achieved satisfactory discriminative power for predicting sarcopenia in populations with healthy controls, chronic obstructive pulmonary disease, and chronic heart failure [170]. While the novel approach enhances the diagnostic accuracy of sarcopenia, its clinical feasibility is limited by the restricted availability and high cost of biomarker measurements in routine practice.

In this regard, pre-dialysis serum creatinine remains the most practical and reliable serum marker for assessing sarcopenia in dialysis patients, as it reflects total skeletal muscle mass. Large-scale observational studies have demonstrated that higher serum creatinine levels, indicative of preserved muscle mass, are associated with better overall survival in HD and PD patients [172,173]. Additionally, to enhance the clinical utility of serum creatinine in practice, several well-validated equations have been developed to estimate lean body mass based on serum creatinine and other clinical variables [165,174,175].

## 6. Interventions and Therapeutic Strategies

**Figure 3:**
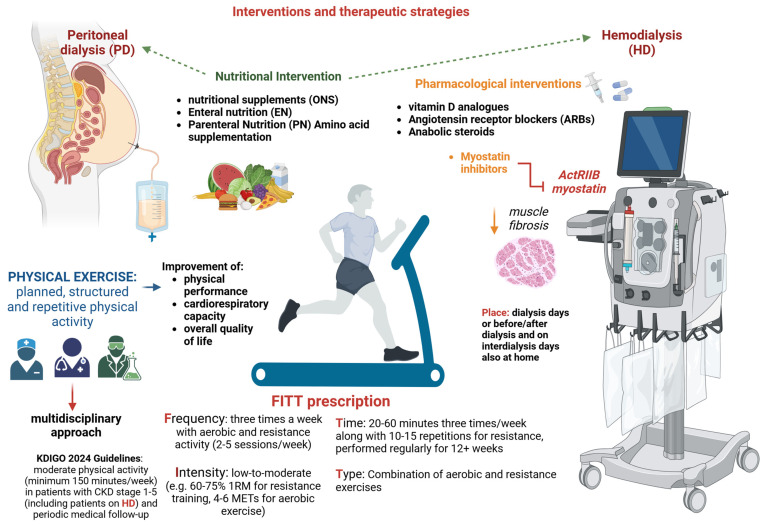
Possible interventions and therapeutic approaches for improving sarcopenia in dialysis patients. These could be categorized into pharmacological and nutritional interventions and tailored physical exercise.

Effective treatment is essential to slow the decline in muscle strength and mass, preserving the ability to perform routine daily activities. Currently, several therapeutic approaches are available for managing sarcopenic patients on dialysis, such as physical exercise, nutritional interventions, and pharmaceutical therapy (Figure 3). A combination of these treatments, rather than any single modality, is currently recommended for the HD population [6].

### 6.1. Physical Exercise

In CKD patients, physical activity levels progressively decline, reaching the nadir with the onset of dialysis [176]. A significant percentage of HD patients have sedentary behavior, which contributes to increased fatigue and frailty [177]. To counteract these adverse effects of physical inactivity, engaging in exercise training is fundamental for maintaining adequate strength, muscle mass, and physical function [178]. Additionally, exercise improves physical performance, cardiorespiratory capacity, and overall quality of life, acting not only as a direct physiological anabolic stimulus for skeletal muscle but also indirectly by modulating the neuroendocrine system [179]. According to Kopple et al., exercise, whether endurance or aerobic training, promotes muscle anabolism and improves lean body mass as well as ensuring exercise adherence [180,181].

It is worth noting that exercise differs from physical activity, as it is a planned, structured, repetitive form of physical activity. Therefore, while nephrologists should encourage HD patients to increase their physical activity levels, exercise prescription requires a multidisciplinary approach. This involves the nephrologist, dialysis nursing staff, dietician, and exercise experts (i.e., physiologists, kinesiologists, or physical therapists) [177]. Ideally, each dialysis center should have a dedicated healthcare team to develop personalized exercise programs that promote adherence and ensure effectiveness [182].

The 2024 KDIGO guidelines recommend physical activity for patients with CKD stages 1–5 (including those on HD), suggesting moderate-intensity physical activity for a minimum of 150 min per week based on their cardiovascular tolerance [183]. Although the risk of cardiovascular events during exercise training has been hypothesized in HD patients, no cardiac events have been reported in any published exercise training studies [177]. However, HD patients should undergo a medical evaluation by sports medicine physicians before beginning an exercise training program. Moreover, KDIGO guidelines do not provide practical guidance on the frequency, type, or location of exercise for HD patients. Instead, exercise prescription should adhere to the FITT approach: Frequency, Intensity, Time, and Type [184].

*Frequency*: Studies suggest exercise for HD patients three times a week in both aerobic and resistance activity, with a median range of two to five sessions per week [185,186]. A randomized controlled pilot efficacy study by Kirkman et al. investigated the impact of progressive resistance training (PRET) on muscle volume, strength, and physical performance to reverse muscle atrophy. Over 12 weeks, high-intensity PRET 3 times a week during hemodialysis elicited an anabolic response and increased strength in HD patients [187]. While these results are promising, the lack of efficacy in improving functional capacity warrants further investigation.

*Intensity*: Several moderate-intensity interdialytic or intradialytic training programs have been reported to be effective at improving muscle strength (60–75% 1 RM for resistance training, 4–6 METs for aerobic exercise) [188], as a surrogate for sarcopenia. Nevertheless, the largest multicenter randomized controlled trial, EXCITE (EXercise Introduction To Enhance Performance in Dialysis), enrolled 296 patients on hemodialysis or peritoneal dialysis who engaged in low-intensity walking activity [189,190]. After 6 months of walking for 20 min 3 times per day, a statistically significant improvement in the active group was found for physical performance, measured by the 6-min walk test (6 MWT) (*p* < 0.001), and for muscle strength, assessed by the 5-times sitting test (5-STS) (*p* = 0.001) [191]. It is worth noting that the benefits of walking training on the 5-STS test were not sustained over an extended period [192].

*Time*: Current evidence suggests that aerobic exercise training should last 20–60 min three times a week, along with one set of 10–15 repetitions for resistance exercise training [193]. Nonetheless, a reduced time of daily 10-min exercise sessions has proven effective for the most frail HD patients [185]. It is important to note that the exercise must be carried out regularly for more than 12 weeks to observe beneficial effects on muscle function [177].

*Type*: Data from RCTs conducted in sarcopenic HD patients suggest that resistance exercise programs should be preferred to enhance strength and promote muscle mass [194]. These resistance exercise programs typically encompass upper and lower limb training using dumbbells, elastic belts, and balls. However, combined (aerobic plus resistance) exercise may be more effective for improving overall physical function in sarcopenic HD patients. In this regard, a recent meta-analysis [194] showed that combined aerobic and resistance exercise improved the peak oxygen uptake, six-minute walk test, and 60 s sit-to-stand test. This may be because resistance training demands a minimum level of cardiopulmonary capacity, which can only be achieved through aerobic exercise [195].

Regarding the *exercise location*, it can be performed on dialysis days (intradialytic or immediately pre/post-dialysis sessions) or interdialytic days, either in the dialysis facility or at home. A recent meta-analysis [196] of 12 RCTs compared the efficacy of home-based exercise programs with intradialytic programs or with usual care in 791 patients on maintenance dialysis (hemodialysis or peritoneal dialysis). Home-based training was found to be superior to usual care but comparable to intradialytic exercise in terms of walking speed (6 MWT; nine RCTs; pooled weighted mean differences (WMDs): 33.7 m, 95% confidence interval (CI): 22.8–44.5; *p* < 0.001; I^2^ = 0%) and aerobic capacity (VO^2^ peak; three RCTs; pooled WMD: 2.04 mL/kg/min, 95% CI: 0.25–3.83; *p* = 0.03; I^2^ = 0%). By contrast, no significant improvement in muscle strength measured by hand grip was observed in home-based exercise programs (two RCTs; pooled WMD 1.06 kg, 95% CI: 2.23–4.35; Z-test = 0.63, *p* = 0.53; I^2^ = 0%). However, the muscle strength was analyzed in only two RCTs, encompassing 36 patients and conducted over 12–16 weeks. Although further research is required to provide more definitive insights, the decision between intradialytic and home-based exercise training should be guided by the available local resources and patient conditions.

### 6.2. Nutritional Intervention

Several nutritional guidelines for dialysis patients have been published (Table 3). Yet, only 50% of them achieve adequate protein and energy intake [197], which may contribute to the development of sarcopenia.

Few studies investigated the impact of nutrition on sarcopenia, particularly in PD patients [180]. Several nutritional strategies, including oral nutritional supplements (ONSs), enteral nutrition (EN), parenteral nutrition (PN), amino-acids supplementation, and other specific nutritional products, have been implemented to improve the protein–energy intake in sarcopenic patients on HD (Table 4). Matsuzawa et al. conducted a meta-analysis of four studies exploring the effect of protein supplementation (oral or parenteral nutrition) on muscle mass, muscle strength, and physical function in patients undergoing HD. Their findings showed that protein supplementation yielded benefits in terms of physical performance but did not influence muscle mass or strength. No studies of a combination of nutritional therapy and exercise were included [198]. It is important to emphasize that nutrition alone does not improve muscle mass and strength unless associated with exercise. The best effects on muscle growth occur when exercise is performed immediately after protein and calorie supplementation [180].

### 6.3. Pharmacological Interventions

In recent years, novel therapeutic interventions, such as vitamin D, angiotensin receptor blockers, inhibitors of myostatin, and anabolic steroids, have been proposed for counteracting sarcopenia in patients undergoing dialysis. Vitamin D has been shown to stimulate myogenesis and inhibit myostatin at the level of skeletal muscle, counteracting muscle catabolism [199]. Experimental studies suggest that vitamin D may influence muscle health by acting on calcium homeostasis and activating vitamin D receptors in skeletal muscle [200]. However, findings from studies conducted in HD patients examining the effects of vitamin D supplementation (i.e., cholecalciferol, calcitriol, or paricalcitol) on muscle mass or strength remain inconclusive [201,202,203,204]. Angiotensin II overexpression has been demonstrated to promote muscle catabolism and proteolysis [180]. Lin et al. identified the role of angiotensin receptor blockers (ARBs) in preserving muscle strength among 120 HD patients. ARBs’ use was independently associated with preserved hand-grip strength after adjustment for multiple confounding factors (OR  =  0.25, 95% CI: 0.07–0.93, *p*  =  0.039) [205]. Myostatin is a myokine that promotes muscle degradation and inhibits myogenesis [206]. Few studies have evaluated serum myostatin in HD patients [207]. Esposito et al. found that circulating myostatin may reflect muscle mass content rather than muscle wasting [208]. Additionally, dialysis treatment may influence myostatin levels, even though myostatin is a medium molecule (26 KDa) [209]. The inhibition of the myostatin pathway is emerging as a novel and compelling therapeutic target for managing sarcopenia. In uremic mice, myostatin inhibition has been shown to reduce and prevent muscle fibrosis [210]. This pathway can be targeted by blocking the myostatin receptor (ActRIIB) or directly inhibiting myostatin. In this regard, the results of an ongoing phase 2 study (NCT01958970) using a peptide against myostatin (AMG745/PINTA745) in HD patients are expected to provide valuable new insights [210].

Finally, as briefly alluded to before, anabolic steroids have been demonstrated to stimulate muscle growth in HD patients. Johansen et found that treatment with nandrolone decanoate for 6 months significantly enhanced lean body mass compared with a placebo group (mean change [SD], +4.5 [2.3] kg; *p* < 0.001 compared with baseline) [88,211]. However, the use of anabolic steroids is limited by their side effects, including erectile dysfunction, gynecomastia, and an increased cardiovascular risk [212,213]. Further RCTs are necessary to evaluate the efficacy and safety of anabolic steroids in addressing sarcopenia among HD patients.

## 7. Future Perspectives

Sarcopenia remains an often-underestimated complication in dialysis patients despite its profound impact on their clinical outcomes, quality of life, and overall prognosis. Its underdiagnosis and limited clinical attention delay timely intervention, leaving many patients vulnerable to preventable disability, reduced functionality, and psychological distress. To address this gap, increasing awareness and stressing clinical importance and the need for integrated management strategies of sarcopenia among nephrologists is of paramount importance.

Greater emphasis on sarcopenia as a critical aspect of dialysis care begins with improved diagnostic approaches. Advances in imaging technologies like ultrasound and bioimpedance analysis provide opportunities for the early and precise identification of muscle wasting. These tools can help overcome the limitations of traditional diagnostic methods, which are often confounded by fluid imbalances in dialysis patients. However, the lack of standardized protocols, the absence of current data in all ethnicities, and disease-specific diagnostic thresholds limit their broader adoption. Future research must focus on refining these modalities and establishing ESKD-specific criteria that can be universally applied.

Beyond diagnosis, the management of sarcopenia requires a holistic and multidisciplinary approach that integrates nutritional support, physical rehabilitation, and pharmacological therapies. Nutritional strategies such as tailored dietary interventions can play a pivotal role in counteracting muscle wasting. However, nutrition alone fails to improve outcomes unless combined with structured exercise programs. Intradialytic exercise interventions, which incorporate physical activity into dialysis sessions, offer a practical solution to overcome time constraints while improving muscle mass, strength, and overall physical function. Such programs deserve further exploration to refine their protocols and maximize adherence.

In parallel, emerging pharmacological therapies hold promise for the treatment of sarcopenia, but the evidence remains too sparse for implementing these in daily practice.

Equally important is addressing the psychosocial impact of sarcopenia, which extends beyond physical disability to encompass anxiety, depression, and social isolation. The interplay between physical and psychological factors in this population underscores the need for an integrative care model that incorporates mental health support alongside physical and nutritional therapies.

## 8. Conclusions

Nephrologists must adopt a more multidisciplinary approach, involving nephrologists, dietitians, physiatrists, and mental health professionals. Increasing awareness of sarcopenia among healthcare providers through education and training programs is equally critical, as it can facilitate earlier recognition and intervention. Policy changes and resource allocation are also necessary to implement exercise programs, expand access to innovative therapies, and support research initiatives that address the unique challenges of sarcopenia in dialysis patients.

## Figures and Tables

**Table 1:** Summary of the main studies providing epidemiological data on sarcopenia in dialysis patients.

Author/Year	Study Type	Population	Findings	Notes
Isoyama et al., 2014 [10]	Post hoc cross-sectional analysis with prospective follow-up	Incident dialysis patients N = 330 Gender: 62% men Mean age: 53 ± 13 years	Sarcopenia prevalence: 20%. Low muscle mass prevalence: 24%. Low muscle strength prevalence: 15%. Multivariable analysis: increased sarcopenia risk associated with old age, low albumin, PEW.	Sarcopenia definition: DXA, MAMC, HGS, EWGS. Old age, comorbidities, PEW, physical inactivity, low albumin, and inflammation associated with low muscle strength but not with low muscle mass.
Lamarca et al., 2014 [11]	Multicenter observational and cross-sectional study	Maintenance HD patients (Rio de Janeiro, Brazil) N = 102 Age > 60 years old Gender: 73.5% men Mean age: 70.7 ± 7 years Median dialysis vintage: 2.25 (1; 5.3)	Wide prevalence of sarcopenia, depending on method and cut-off. Sarcopenia prevalence: 4–63%. Decreased muscle mass prevalence: 4–73.5%. Decreased muscle strength prevalence: 85%. Sarcopenia prevalence by >2 criteria: 2–15%.	Sarcopenia definition: one criterion for low muscle mass (DXA, BIA, sum of SKF, calf circumference, and MAMC) and one for low muscle strength. Comparable agreement between DXA, BIA, and SKF. No gait speed available.
Wathanavasin et al., 2022 [14]	Systematic review and meta-analysis	Dialysis patients N = 7576 Studies N = 41 (31 in HD, 7 in PD, 3 in HD + PD population) Mean age: 62.3 years Gender: 61.4% men Five continents: Asia 45.6%; Europe 25.9%; North America 14.3%; South America 13.7%; Australia: 0.5% Mean dialysis vintage: 52.4 months	Pooled sarcopenia prevalence: 25.6% (95% CI: 22.1% to 29.4%). Regional sarcopenia prevalence: 15.4% in the USA, 17.9% in Australia, 20.4% in South America, 27.9% in Asia, 29.1% in Europe. Sarcopenia prevalence by diagnostic criteria: 36.9% by AWGS 2019, 34.9% by IWGS, 24.4% by EWGSOP 2010, 24.1% by EWGSOP 2019, 22% by AWGS 2014, 20% by FNIH. Sarcopenia prevalence by dialysis modality: 26.8% in HD, 17.5% in PD.	Sarcopenia definition: both low muscle mass and low muscle strength. Substantial heterogeneity (I2 = 91.98%, *p* < 0.001). Higher risk of sarcopenia in men and diabetics.
Duarte et al., 2024 [15]	Systematic review and meta-analysis	Studies N = 140 (42,041 patients, 25 countries, 5 continents) Sarcopenia prevalence: CKD + dialysis patients (114 studies) N = 36,190 HD patients (63 studies) N = 18,190 PD patients (8 studies) N = 1283 HD + PD patients (4 studies) N = 662	Sarcopenia prevalence in dialysis patients: 27.7 (95% CI: 24.7–30.9). Severe sarcopenia prevalence in dialysis patients: 26.2% (95% CI: 16.6–37.1). Similar sarcopenia prevalence between dialysis and non-dialysis patients. Significantly higher frequency of severe sarcopenia in dialysis vs. non-dialysis patients.	Sarcopenia definition: EWGSOP; EWGSOP2; IWGS; AWGS; AWGS 2019; FNIH. Sarcopenia traits prevalence in dialysis: 50% for low muscle strength, 32.2% for low muscle mass, 46.8% (HD), and 47.5% (PD) for low physical performance. Higher frequency of low muscle strength in dialysis vs. non-dialysis patients.
Shu et al., 2022 [7]	Systematic review and meta-analysis	Dialysis patients: N = 6162 Studies N = 30 (20 in the HD population and 10 in the PD population; 14 in Asia, 8 in Europe, and 8 in America) Mean age: 47.5 to 77.5 years; mean dialysis vintage: 3 to 91.7 months	Overall sarcopenia prevalence: 28.5% (95% CI: 22.9–34.1%). Sarcopenia prevalence by combined criteria: 25.9% (95% CI: 20.4–31.3%). Higher sarcopenia prevalence in HD (31%) vs. PD populations (23.4%). Higher sarcopenia prevalence in studies using low muscle mass only (34.6%) vs. those using combined criteria (25.9%). Lower sarcopenia prevalence by EWGSOP criteria (23.4%) vs. AWGS criteria (42.6%) and other criteria (32.2%).	Sarcopenia definition: Low muscle mass plus low muscle strength and/or low physical performance (22 studies); low muscle mass (8 studies); EWGSOP criteria (17 studies); AWGS criteria (4 studies). No effects of age and dialysis duration on prevalence. No significant differences between dialysis modalities, diagnostic criteria, and consensuses.
Duarte et al., 2024 [16]	Multicenter, cross-sectional	HD patients (Brazil) N = 838 Gender: 61% men Mean age: 57.8 ± 15.0 years	Sarcopenia prevalences are similar between consensuses: 15.3% (128 patients) by EWGSOP2; 12.5% (105 patients) by SDOC. Low muscle strength: 52.3% by SDOC vs. 25.9% by EWGSOP2. Agreement between consensuses: Weak (50 of 233 patients, 21.5%; κ = 0.34, 95% CI: 0.25–0.43).	Sarcopenia definition: EWGSOP2 and SDOC. Marginally better agreement for older patients.
Hung et al., 2017 [17]	Retrospective analysis, cross-sectional	PD patients N = 325 Gender: 57.2% men Mean age: 56.7 ± 16.5 years Ethnicity: White, Asian, African/Afro-Caribbean	Sarcopenia prevalence by gender: 2.2–31.3% for women 25.1–75.6% for men Greater sarcopenia prevalence for men by all grading systems. No effects of diabetes, ethnicity, or dialysis adequacy on prevalence.	Sarcopenia definition: DXA. No muscle strength measurements. Male patients older (58.3 ± 16) compared with women (53.4 ± 15.7 years). Increased sarcopenia prevalence in PD patients vs. age-matched subjects by ALM sex-specific cut-offs from healthy young adults.
Yoowannakul et al., 2018 [18]	Cross-sectional	HD patients N = 600 Gender: 62.2% men Mean age 66.3 ± 14.7 Ethnicity: White, N = 281; Asian, N = 167; Black, N = 149; Unclassified, N = 3	Similar muscle strength between ethnicities: AWGS: 80% Asian vs. 70% White vs. 64%; Black; EWGS: 90% Asian vs. 77.5% White vs. 76% Black; FNIH: 70% Asian vs. 62.5% White vs. 40% Black. Lower muscle mass in Asian vs. White and Black: AWGS: 45% Asian vs. 25% White vs. 8%, Black; EWGS: 45% Asian vs. 25% White vs. 8% Black; FNIH: 55% Asian vs. 25% White vs. 8% Black	Sarcopenia definition: HGS, multifrequency BIA; FNIH, EWGS, AWGS. Muscle weakness more common than reduced muscle mass. Increased prevalence of low muscle mass in Asians after adjusting for height.
Yoowannakul et al.; 2018 [19]	Cross-sectional	PD patients N = 434 Gender: 55.1% men Mean age: 55.3 ± 16.2 years Ethnicity: White N = 235; Black N = 83; Asian N = 113; other ethnicities N = 13	Sarcopenia prevalence: 6.5–26.3%. Greater sarcopenia prevalence in men by EWG, FHIN. Greater sarcopenia prevalence in Asians > 40% by EWG; >35% by FNIH; vs. White (2.3–18.7%), Black (3.8–15.7%) by EWG and FNIH. Sarcopenia prevalence in Asians < 11% by AWGS cut-off points.	Sarcopenia definition: BIA; ESWGOP, FHIN, AWGS. No association between sarcopenia prevalence and residual renal function, serum albumin, CRP, or co-morbidity score.
Ren et al.; 2016 [20]	Cross-sectional	HD patients N = 131 Gender: 81.1% men Mean age: 49.4 ± 11.7	Sarcopenia prevalence: 13.7%. Gender-related sarcopenia prevalence: 5% in men, 11.8% in women. Age-related sarcopenia prevalence: 18.0% in patients > 50 years; 33.3% in patients > 60 years.	Sarcopenia definition: BIA, HGS, EWGSOP. Independent sarcopenia risk factors in multivariate analysis: dialysis duration, diabetes, serum phosphorus.

Legend: AWGS, Asian Working Group for Sarcopenia; BIA, bioelectrical impedance; DXA, dual-energy X-ray absorptiometry; EWGSOP, European Working Group on Sarcopenia in Older People; FNIH, Foundation for the National Institutes of Health Sarcopenia project; HGS, hand-grip strength; HD, hemodialysis; IWGS, International Working Group on Sarcopenia; MAMC, mid-arm muscle circumference; PD, peritoneal dialysis; PEW, protein energy wasting; SDOC, Sarcopenia Definitions and Outcomes Consortium; SKF, skinfold thicknesses.

**Table 2:** Main clinical studies evaluating the impact of sarcopenia risk measures on clinical outcomes.

Study	Year	Study Design	Sample Size (n)	Main Findings
Abdala et al. [110]	2021	Cross-sectional	100	High prevalence of falls in patients with lower HGS.
Alston et al. [99]	2018	Cross-sectional	113	Association between appendicular lean mass (ALM) index and increased self-reported depression, anxiety, and decreased general health.
Baltac et al. [108]	2022	Prospective cohort	106	No association between sarcopenia and pre-atherosclerotic markers, cardiovascular events, and all-cause mortality.
Chao Li et al. [111]	2021	Cross-sectional	112	Severe sarcopenia was independently associated with anorexia.
Cheng et al. [103]	2021	Cross-sectional	238	Severe sarcopenia was significantly associated with dependency in the basic and instrumental activities of daily living (ADLs) (OR, 4.68, 95% CI: 2.11–10.40; OR, 3.24, 95% CI: 1.61–6.53, respectively).
Elder et al. [112]	2023	Prospective cohort	77	Sarcopenia is highly prevalent in elderly hemodialysis patients but is not an independent predictor of mortality.
Ferreira et al. [113]	2022	Cross-sectional	127	Patients diagnosed with sarcopenia had almost three times higher risk for mortality.
Giglio et al. [104]	2018	Cross-sectional	170	In the adjusted multivariate Cox analysis, low muscle strength and sarcopenia were associated with higher hospitalization rates. Sarcopenia was a predictor of mortality.
Heeryong Lee et al. [114]	2020	Cross-sectional	131	Inadequate nutrition was associated with the risk of osteoporosis and sarcopenia but not cognitive impairment.
Hiroya Hayashi et al. [115]	2022	Retrospective cohort	244	Both sarcopenia and dynapenia resulted in significantly higher CV events than non-sarco-dynapenia in patients undergoing HD (HR 8.00; 95% CI: 2.73–34.1; *p* < 0.0001 vs. HR 4.85; 95% CI: 1.28–23.0; *p* < 0.02).
Hyung Eun Son et al. [116]	2022	Cross-sectional	177	Low skeletal muscle mass to dry body weight ratio (SMM/WT) had a higher rate of intradialytic hypotension (40.7%).
Ishimura et al. [117]	2022	Retrospective cohort	308	Patients with sarcopenia and sarcopenic obesity had significantly higher rates of all-cause mortality (*p* = 0.0004).
Isoyama et al. [10]	2014	Cross-sectional	330	Low muscle strength was more strongly associated with aging, protein-energy wasting, physical inactivity, inflammation, and mortality.
de Luca Corrêa et al. [118]	2023	Prospective study	247	Sarcopenic patients had higher numbers of cardiovascular disease (56.9% vs. 12.6%) and hospitalizations (93.8% vs. 49.5%). Sarcopenia was associated with a significantly higher risk of mortality (HR = 3.3, 95% CI: 1.6–6.9, *p* = 0.001).
Kittiskulnam et al. [119]	2017	Prospective cohort	645	Both gait slowness and low hand-grip strength significantly improved the net reclassification index compared with models without performance measures (50.5% for slowness and 33.7% for weakness).
Kobayashi et al. [120]	2021	Cross-sectional	58	The Skeletal Muscle Mass Index (SMI) and Geriatric Nutritional Risk Index (GNRI) were the factors associated with all-cause mortality in all patients.
Kono et al. [121]	2021	Prospective cohort	635	Hand-grip strength (HR 3.61, 95% CI: 1.70–7.68, *p* < 0.001) and the five-times chair stand test (HR 1.71 95% CI: 1.01–2.90, *p* = 0.045) were significant predictors for mortality.
Mori et al. [122]	2019	Prospective cohort	308	Patients with sarcopenia demonstrated significantly higher rates of all-cause mortality.
Ren et al. [20]	2016	Cross-sectional	131	The one-year survival in sarcopenic patients (88.9%) was significantly lower than that in non-sarcopenic patients.
Sánchez-Tocino et al. [123]	2022	Prospective cohort	60	Appendicular skeletal muscle mass (ASM) and severity (gait speed, GS) variables were associated with mortality (HR 3.03, 95% CI: 1.14–8.08, *p* = 0.028).
Ting-Yun Lin et al. [124]	2020	Cross-sectional	263	Low appendicular skeletal muscle mass predicted by the Body Composition Monitor (BCM) equation was associated with significantly worse overall survival among CKD patients but not those on chronic hemodialysis.
Tsujimoto et al. [125]	2024	Prospective cohort	450	Concomitant sarcopenia and malnutrition were significantly associated with a risk of mortality (HR 2.10; 95% CI: 1.05–4.21; *p* = 0.037).
Ulgen et al. [126]	2022	Cross-sectional	79	Decreased skeletal muscle mass contributes to increased arterial stiffness in hemodialysis patients.
Wang et al. [127]	2023	Cross-sectional	130	Sarcopenia was associated with Low Bone Mineral Disease (BMD) (OR = 5.894, 95% CI: 1.592–21.830, *p* < 0.01).
Xavier et al. [128]	2022	Cross-sectional	218	Worse nutritional status increases the risk of lower hand-grip strength and mortality in hemodialysis patients.
Xiang et al. [129]	2023	Prospective cohort	209	Osteosarcopenia was independently associated with all-cause mortality (HR = 3.74, 95% CI: 1.172–11.938), while osteoporosis alone and sarcopenia alone were not.
Yang et al. [130]	2023	Prospective cohort	1117	Both greater changes in the appendicular skeletal mass index and hand-grip strength had a lower risk of cognitive impairment (adjusted OR = 0.857, 95% CI: 0.778–0.945, *p* = 0.002; adjusted OR = 0.976, 95% CI: 0.963–0.989, *p* < 0.001, respectively).
Yoshikoshi et al. [131]	2024	Retrospective cohort	328	Osteosarcopenia showed a higher risk of all-cause mortality than the robust group.
Yoshikoshi et al. [132]	2022	Retrospective cohort	616	Dynapenia was associated with increased risks of all-cause mortality and CV hospitalizations among patients on hemodialysis.
Yu Ho Lee et al. [133]	2020	Prospective cohort	277	Patients with low gait speed and hand-grip strength had the highest risks for all-cause mortality and cardiovascular events among the groups (adjusted HR 2.72, *p* = 0.024).
Yuenyongchaiwat et al. [134]	2021	Cross-sectional	104	Sarcopenic patients had low physical activity, a high depression score, and an increased mortality risk.
Yu-Li Lin et al. [105]	2020	Cross-sectional	126	Muscle quality (HR = 0.42, 95% CI: = 0.19–0.93, *p* = 0.032) was independently associated with the composite outcome of hospitalization or death.
Zhou et al. [135]	2023	Cross-sectional	2743	The association between sarcopenic obesity and cognitive impairment was statistically significant after adjusting for age, sex, and educational status (OR, 1.47; 95% CI: 1.11–1.96).

Legend: CI, confidence interval; CV, cardiovascular; CKD, chronic kidney disease; HD, hemodialysis; HR, hazard ratio; NR, not reported; OR, odds ratio.

**Table 3:** Recommendations from the main guidelines for protein and energy intake in patients on hemodialysis and peritoneal dialysis.

Guidelines	Hemodialysis	Peritoneal Dialysis
ESPEN (2006)	Protein intake: 1.2–1.4 g/kg/day Energy intake: 35 kcal/kg/day	Protein intake: 1.2–1.5 g/kg/day Energy intake: 35 kcal/kg/day
EBPG (2007)	Protein intake: >1.1 g/kg/day Energy intake: 30–40 kcal/kg/day	Protein intake: ≥1.2 g/kg/day Energy intake: 35 kcal/kg/day
ISRNM (2013)	Protein intake: >1.2 g/kg/day Energy intake: 30 kcal/kg/day (≥60 years) 35 kcal/kg/day (<60 years)	Protein intake: >1.2 g/kg/day Energy intake: 30–35 kcal/kg/day (including dialysate)
KDOQI (2020)	Protein intake: 1–1.2 g/kg/day Energy intake: 25–35 kcal/kg/day	Protein intake: 1–1.2 g/kg/day Energy intake: 25–35 kcal/kg/day

Legend: EBPG, European Best Practice Guideline; ESPEN, European Society for Clinical Nutrition and Metabolism; KDOQI, Kidney Disease Outcomes Quality Initiative; ISRNM, International Society of Renal Nutrition and Metabolism.

**Table 4:** Recommendations for starting oral nutrition supplements, enteral nutrition, or parenteral nutrition.

Type of Nutrition	Indication
ONS	Poor appetite and failure in spontaneous dietary intake Energy intake < 30 kcal/kg/day and protein intake < 1.2 g/kg/day * Serum albumin < 3.8 g/dL or prealbumin < 28 mg/mL Unintentional weight loss > 5% Diagnosis of PEW using SGA definition
EN	Patients unable to tolerate nutritional supplementation by mouth and failing on dietary intake with ONS Severe PEW Energy intake < 20 kcal/kg/day Undergoing metabolic stress
PN	If all other forms of nutrition failed ^ For patients with spontaneous intake of at least 20 kcal/kg/day and protein intake of 0.8 g/kg/day

Legend: EN, enteral nutrition; ONS, oral nutrition supplement; PEW, protein-energy wasting; PN, parenteral nutrition; SGA, subjective global assessment; * as per ISRNM guidelines; ^ as per EBPG and KDOQI guidelines, while according to ESPEN guidelines, intradialytic parenteral nutrition should be started before starting EN.

## Data Availability

No new data were created or analyzed in this study.

## References

[1] Jadoul M., Aoun M., Masimango Imani M. (2024). The major global burden of chronic kidney disease. Lancet Glob. Health.

[2] Jankowski J., Floege J., Fliser D., Bohm M., Marx N. (2021). Cardiovascular Disease in Chronic Kidney Disease: Pathophysiological Insights and Therapeutic Options. Circulation.

[3] van Oevelen M., Bonenkamp A.A., van Eck van der Sluijs A., Bos W.J.W., Douma C.E., van Buren M., Meuleman Y., Dekker F.W., van Jaarsveld B.C., Abrahams A.C. (2024). Health-related quality of life and symptom burden in patients on haemodialysis. Nephrol. Dial. Transplant..

[4] Vanden Wyngaert K., Van Craenenbroeck A.H., Eloot S., Calders P., Celie B., Holvoet E., Van Biesen W. (2020). Associations between the measures of physical function, risk of falls and the quality of life in haemodialysis patients: A cross-sectional study. BMC Nephrol..

[5] Kirk B., Cawthon P.M., Arai H., Avila-Funes J.A., Barazzoni R., Bhasin S., Binder E.F., Bruyere O., Cederholm T., Chen L.K. (2024). The Conceptual Definition of Sarcopenia: Delphi Consensus from the Global Leadership Initiative in Sarcopenia (GLIS). Age Ageing.

[6] Sayer A.A., Cooper R., Arai H., Cawthon P.M., Ntsama Essomba M.J., Fielding R.A., Grounds M.D., Witham M.D., Cruz-Jentoft A.J. (2024). Sarcopenia. Nat. Rev. Dis. Primers.

[7] Shu X., Lin T., Wang H., Zhao Y., Jiang T., Peng X., Yue J. (2022). Diagnosis, prevalence, and mortality of sarcopenia in dialysis patients: A systematic review and meta-analysis. J. Cachexia Sarcopenia Muscle.

[8] Ertuglu L., Ikizler T.A. (2024). Nutrition Management in Geriatric Patients with CKD. Kidney360.

[9] Shafiee G., Keshtkar A., Soltani A., Ahadi Z., Larijani B., Heshmat R. (2017). Prevalence of sarcopenia in the world: A systematic review and meta- analysis of general population studies. J. Diabetes Metab. Disord..

[10] Isoyama N., Qureshi A.R., Avesani C.M., Lindholm B., Bàràny P., Heimbürger O., Cederholm T., Stenvinkel P., Carrero J.J. (2014). Comparative associations of muscle mass and muscle strength with mortality in dialysis patients. Clin. J. Am. Soc. Nephrol..

[11] Lamarca F., Carrero J.J., Rodrigues J.C., Bigogno F.G., Fetter R.L., Avesani C.M. (2014). Prevalence of sarcopenia in elderly maintenance hemodialysis patients: The impact of different diagnostic criteria. J. Nutr. Health Aging.

[12] Ter Beek L., Vanhauwaert E., Slinde F., Orrevall Y., Henriksen C., Johansson M., Vereecken C., Rothenberg E., Jager-Wittenaar H. (2016). Unsatisfactory knowledge and use of terminology regarding malnutrition, starvation, cachexia and sarcopenia among dietitians. Clin. Nutr..

[13] Carrero J.J., Johansen K.L., Lindholm B., Stenvinkel P., Cuppari L., Avesani C.M. (2016). Screening for muscle wasting and dysfunction in patients with chronic kidney disease. Kidney Int..

[14] Wathanavasin W., Banjongjit A., Avihingsanon Y., Praditpornsilpa K., Tungsanga K., Eiam-Ong S., Susantitaphong P. (2022). Prevalence of Sarcopenia and Its Impact on Cardiovascular Events and Mortality among Dialysis Patients: A Systematic Review and Meta-Analysis. Nutrients.

[15] Duarte M.P., Almeida L.S., Neri S.G.R., Oliveira J.S., Wilkinson T.J., Ribeiro H.S., Lima R.M. (2024). Prevalence of sarcopenia in patients with chronic kidney disease: A global systematic review and meta-analysis. J. Cachexia Sarcopenia Muscle.

[16] Duarte M.P., Nobrega O.T., Baiao V.M., Vieira F.A., Monteiro J.S., Pereira M.S., Pires L.F., Queiroz G.G., Silva M.J., Silva M.Z.C. (2024). Agreement between the EWGSOP2 and SDOC consensuses for sarcopenia in patients receiving hemodialysis: Findings of a cross sectional analysis from the SARC-HD study. Nutr. Clin. Pract..

[17] Hung R., Wong B., Goldet G., Davenport A. (2017). Differences in Prevalence of Muscle Wasting in Patients Receiving Peritoneal Dialysis per Dual-Energy X-Ray Absorptiometry Due to Variation in Guideline Definitions of Sarcopenia. Nutr. Clin. Pract..

[18] Yoowannakul S., Tangvoraphonkchai K., Vongsanim S., Mohamed A., Davenport A. (2018). Differences in the prevalence of sarcopenia in haemodialysis patients: The effects of gender and ethnicity. J. Hum. Nutr. Diet..

[19] Yoowannakul S., Tangvoraphonkchai K., Davenport A. (2018). The prevalence of muscle wasting (sarcopenia) in peritoneal dialysis patients varies with ethnicity due to differences in muscle mass measured by bioimpedance. Eur. J. Clin. Nutr..

[20] Ren H., Gong D., Jia F., Xu B., Liu Z. (2016). Sarcopenia in patients undergoing maintenance hemodialysis: Incidence rate, risk factors and its effect on survival risk. Ren. Fail..

[21] Kelly T.L., Wilson K.E., Heymsfield S.B. (2009). Dual energy X-Ray absorptiometry body composition reference values from NHANES. PLoS ONE.

[22] Davenport A., Hussain Sayed R., Fan S. (2011). The effect of racial origin on total body water volume in peritoneal dialysis patients. Clin. J. Am. Soc. Nephrol..

[23] Lee S.W., Song J.H., Kim G.A., Lee K.J., Kim M.J. (2001). Assessment of total body water from anthropometry-based equations using bioelectrical impedance as reference in Korean adult control and haemodialysis subjects. Nephrol. Dial. Transplant..

[24] Chen L.K., Liu L.K., Woo J., Assantachai P., Auyeung T.W., Bahyah K.S., Chou M.Y., Chen L.Y., Hsu P.S., Krairit O. (2014). Sarcopenia in Asia: Consensus Report of the Asian Working Group for Sarcopenia. J. Am. Med. Dir. Assoc..

[25] Crystal F., Fulai R., Kaonga P., Davenport A. (2024). Malnutrition, protein energy wasting and sarcopenia in patients attending a haemodialysis centre in sub-Saharan Africa. Eur. J. Clin. Nutr..

[26] Aucella F., Battaglia Y., Bellizzi V., Bolignano D., Capitanini A., Cupisti A. (2015). Physical exercise programs in CKD: Lights, shades and perspectives. J. Nephrol..

[27] Torino C., Manfredini F., Bolignano D., Aucella F., Baggetta R., Barilla A., Battaglia Y., Bertoli S., Bonanno G., Castellino P. (2014). Physical performance and clinical outcomes in dialysis patients: A secondary analysis of the EXCITE trial. Kidney Blood Press. Res..

[28] Musolino M., Presta P., Cianfrone P., Errante M.A., Andreucci M., Coppolino G., Bolignano D. (2023). Self-Reported Physical Inactivity and Mood Disturbances in End-Stage Kidney Disease (ESKD) Patients on Chronic Dialysis Treatment. J. Clin. Med..

[29] Manfredini F., Lamberti N., Malagoni A.M., Felisatti M., Zuccala A., Torino C., Tripepi G., Catizone L., Mallamaci F., Zoccali C. (2015). The role of deconditioning in the end-stage renal disease myopathy: Physical exercise improves altered resting muscle oxygen consumption. Am. J. Nephrol..

[30] Deger S.M., Hung A.M., Gamboa J.L., Siew E.D., Ellis C.D., Booker C., Sha F., Li H., Bian A., Stewart T.G. (2017). Systemic inflammation is associated with exaggerated skeletal muscle protein catabolism in maintenance hemodialysis patients. JCI Insight.

[31] Visser W.J., Egmond A., Timman R., Severs D., Hoorn E.J. (2020). Risk Factors for Muscle Loss in Hemodialysis Patients with High Comorbidity. Nutrients.

[32] Kalantar-Zadeh K., Kopple J.D. (2001). Relative contributions of nutrition and inflammation to clinical outcome in dialysis patients. Am. J. Kidney Dis..

[33] Kaysen G.A., Dubin J.A., Muller H.G., Mitch W.E., Rosales L.M., Levin N.W. (2002). Relationships among inflammation nutrition and physiologic mechanisms establishing albumin levels in hemodialysis patients. Kidney Int..

[34] Kaizu Y., Kimura M., Yoneyama T., Miyaji K., Hibi I., Kumagai H. (1998). Interleukin-6 may mediate malnutrition in chronic hemodialysis patients. Am. J. Kidney Dis..

[35] Hortegal E.V.F., Alves J., Santos E.J.F., Nunes L.C.R., Galvao J.C., Nunes R.F., Lula D.A., Carvalho S.C.R., Franca A., Santos E.M.D. (2020). Sarcopenia and inflammation in patients undergoing hemodialysis. Nutr. Hosp..

[36] Hsu B.G., Wang C.H., Tsai J.P., Chen Y.H., Hung S.C., Lin Y.L. (2024). Association of serum intact parathyroid hormone levels with sarcopenia in patients undergoing peritoneal dialysis. Front. Med..

[37] Kang S.H., Do J.Y. (2020). Effects of volume status on body composition in incident peritoneal dialysis patients. Eur. J. Clin. Nutr..

[38] Song Y., Zhang Q., Ni L., Zhang M., Wang M., Zhang W., Chen J. (2022). Risk Factors Affecting Muscle Mass Decline in Maintenance Hemodialysis Patients. BioMed Res. Int..

[39] Ikeda M., Honda H., Takahashi K., Shishido K., Shibata T. (2016). N-Terminal Pro-B-Type Natriuretic Peptide as a Biomarker for Loss of Muscle Mass in Prevalent Hemodialysis Patients. PLoS ONE.

[40] Wang M., Liu L., Shen X., Li Y., He Q. (2021). Assessing lean tissue by bioelectrical impedance analysis pre hemodialysis underestimates the prevalence of sarcopenia in maintenance hemodialysis patients. Eur. J. Clin. Nutr..

[41] Molina P., Vizcaino B., Molina M.D., Beltran S., Gonzalez-Moya M., Mora A., Castro-Alonso C., Kanter J., Avila A.I., Gorriz J.L. (2018). The effect of high-volume online haemodiafiltration on nutritional status and body composition: The ProtEin Stores prEservaTion (PESET) study. Nephrol. Dial. Transplant..

[42] Ikizler T.A., Flakoll P.J., Parker R.A., Hakim R.M. (1994). Amino acid and albumin losses during hemodialysis. Kidney Int..

[43] Dulaney J.T., Hatch F.E. (1984). Peritoneal dialysis and loss of proteins: A review. Kidney Int..

[44] Do J.Y., Kim A.Y., Kang S.H. (2021). Peritoneal Protein Loss Is Not Associated with Sarcopenia in Peritoneal Dialysis Patients. Front. Med..

[45] Dai N., Diao Z., Huang H., Li Z., Yang R., Liu W. (2024). Disturbed carnitine metabolism is independently correlated with sarcopenia and prognosis in patients on hemodialysis. Clin. Nutr..

[46] Takashima H., Maruyama T., Abe M. (2021). Significance of Levocarnitine Treatment in Dialysis Patients. Nutrients.

[47] Siami G., Clinton M.E., Mrak R., Griffis J., Stone W. (1991). Evaluation of the effect of intravenous L-carnitine therapy on function, structure and fatty acid metabolism of skeletal muscle in patients receiving chronic hemodialysis. Nephron.

[48] Vaux E.C., Taylor D.J., Altmann P., Rajagopalan B., Graham K., Cooper R., Bonomo Y., Styles P. (2004). Effects of carnitine supplementation on muscle metabolism by the use of magnetic resonance spectroscopy and near-infrared spectroscopy in end-stage renal disease. Nephron Clin. Pract..

[49] Abboud M., Rybchyn M.S., Liu J., Ning Y., Gordon-Thomson C., Brennan-Speranza T.C., Cole L., Greenfield H., Fraser D.R., Mason R.S. (2017). The effect of parathyroid hormone on the uptake and retention of 25-hydroxyvitamin D in skeletal muscle cells. J. Steroid Biochem. Mol. Biol..

[50] Visser M., Deeg D.J., Lips P. (2003). Low vitamin D and high parathyroid hormone levels as determinants of loss of muscle strength and muscle mass (sarcopenia): The Longitudinal Aging Study Amsterdam. J. Clin. Endocrinol. Metab..

[51] Wang L., Luo Q., Zhu B., Zhou F. (2019). Relation of Serum 25-Hydroxyvitamin D Status with Skeletal Muscle Mass and Grip Strength in Patients on Peritoneal Dialysis. J. Nutr. Sci. Vitaminol..

[52] Hori M., Takahashi H., Kondo C., Hayashi F., Tokoroyama S., Mori Y., Tsujita M., Shirasawa Y., Takeda A., Morozumi K. (2024). Association between Serum 25-Hydroxyvitamin D Levels and Sarcopenia in Patients Undergoing Chronic Haemodialysis. Am. J. Nephrol..

[53] Leng Y.-J., Wang G.-R., Xie R.-N., Jiang X., Li C.-X., Nie Z.-M., Li T. (2024). Risk Prediction Models for Sarcopenia in Dialysis Patients: A Systematic Review. J. Ren. Nutr..

[54] Riche K.D., Arnall J., Rieser K., East H.E., Riche D.M. (2016). Impact of vitamin D status on statin-induced myopathy. J. Clin. Transl. Endocrinol..

[55] Prabhala A., Garg R., Dandona P. (2000). Severe myopathy associated with vitamin D deficiency in western New York. Arch. Intern. Med..

[56] Baker L.R., Ackrill P., Cattell W.R., Stamp T.C., Watson L. (1974). Iatrogenic osteomalacia and myopathy due to phosphate depletion. Br. Med. J..

[57] Zahed N., Chehrazi S., Falaknasi K. (2014). The evaluation of relationship between vitamin D and muscle power by micro manual muscle tester in end-stage renal disease patients. Saudi J. Kidney Dis. Transplant..

[58] Schott G.D., Wills M.R. (1976). Muscle weakness in osteomalacia. Lancet.

[59] Patten B.M., Bilezikian J.P., Mallette L.E., Prince A., Engel W.K., Aurbach G.D. (1974). Neuromuscular disease in primary hyperparathyroidism. Ann. Intern. Med..

[60] Mallette L.E., Patten B.M., Engel W.K. (1975). Neuromuscular disease in secondary hyperparathyroidism. Ann. Intern. Med..

[61] Delbridge L.W., Marshman D., Reeve T.S., Crummer P., Posen S. (1988). Neuromuscular symptoms in elderly patients with hyperparathyroidism: Improvement with parathyroid surgery. Med. J. Aust..

[62] Smogorzewski M., Piskorska G., Borum P.R., Massry S.G. (1988). Chronic renal failure, parathyroid hormone and fatty acids oxidation in skeletal muscle. Kidney Int..

[63] Campistol J.M. (2002). Uremic myopathy. Kidney Int..

[64] Thompson C.H., Kemp G.J., Taylor D.J., Ledingham J.G., Radda G.K., Rajagopalan B. (1993). Effect of chronic uraemia on skeletal muscle metabolism in man. Nephrol. Dial. Transplant..

[65] Giovannetti S., Biagini M., Balestri P.L., Navalesi R., Giagnoni P., De Matteis A., Ferro-Milone P., Perfetti C. (1969). Uraemia-like syndrome in dogs chronically intoxicated with methylguanidine and creatinine. Clin. Sci..

[66] Bennett S.E., Bevington A., Walls J. (1994). Regulation of intracellular creatine in erythrocytes and myoblasts: Influence of uraemia and inhibition of Na, K-ATPase. Cell Biochem. Funct..

[67] Sohn H.J., Stokes G.S., Johnston H. (1992). An Na, K ATPase inhibitor from ultrafiltrate obtained by hemodialysis of patients with uremia. J. Lab. Clin. Med..

[68] Kittiskulnam P., Srijaruneruang S., Chulakadabba A., Thokanit N.S., Praditpornsilpa K., Tungsanga K., Eiam-Ong S. (2020). Impact of Serum Bicarbonate Levels on Muscle Mass and Kidney Function in Pre-Dialysis Chronic Kidney Disease Patients. Am. J. Nephrol..

[69] Visser W.J., van de Braak E.E.M., de Mik-van Egmond A.M.E., van der Burgh A.C., de Roos N.M., Jans I., van der Hoef I., Olieman J.F., Hoorn E.J., Severs D. (2023). Effects of correcting metabolic acidosis on muscle mass and functionality in chronic kidney disease: A systematic review and meta-analysis. J. Cachexia Sarcopenia Muscle.

[70] Mitch W.E., Du J., Bailey J.L., Price S.R. (1999). Mechanisms causing muscle proteolysis in uremia: The influence of insulin and cytokines. Miner. Electrolyte Metab..

[71] Garibotto G., Sofia A., Russo R., Paoletti E., Bonanni A., Parodi E.L., Viazzi F., Verzola D. (2015). Insulin sensitivity of muscle protein metabolism is altered in patients with chronic kidney disease and metabolic acidosis. Kidney Int..

[72] Uribarri J., Levin N.W., Delmez J., Depner T.A., Ornt D., Owen W., Yan G. (1999). Association of acidosis and nutritional parameters in hemodialysis patients. Am. J. Kidney Dis..

[73] Lofberg E., Gutierrez A., Anderstam B., Wernerman J., Bergstrom J., Price S.R., Mitch W.E., Alvestrand A. (2006). Effect of bicarbonate on muscle protein in patients receiving hemodialysis. Am. J. Kidney Dis..

[74] Liakopoulos V., Roumeliotis S., Zarogiannis S., Eleftheriadis T., Mertens P.R. (2019). Oxidative stress in hemodialysis: Causative mechanisms, clinical implications, and possible therapeutic interventions. Semin. Dial..

[75] Kaltsatou A., Sakkas G.K., Poulianiti K.P., Koutedakis Y., Tepetes K., Christodoulidis G., Stefanidis I., Karatzaferi C. (2015). Uremic myopathy: Is oxidative stress implicated in muscle dysfunction in uremia?. Front. Physiol..

[76] Moylan J.S., Reid M.B. (2007). Oxidative stress, chronic disease, and muscle wasting. Muscle Nerve.

[77] Allen D.G., Lamb G.D., Westerblad H. (2008). Skeletal muscle fatigue: Cellular mechanisms. Physiol. Rev..

[78] Lamb G.D., Westerblad H. (2011). Acute effects of reactive oxygen and nitrogen species on the contractile function of skeletal muscle. J. Physiol..

[79] Taes Y.E., Speeckaert M., Bauwens E., De Buyzere M.R., Libbrecht J., Lameire N.H., Delanghe J.R. (2004). Effect of dietary creatine on skeletal muscle myosin heavy chain isoform expression in an animal model of uremia. Nephron Exp. Nephrol..

[80] Lim P.S., Cheng Y.M., Wei Y.H. (2002). Increase in oxidative damage to lipids and proteins in skeletal muscle of uremic patients. Free Radic. Res..

[81] Crowe A.V., McArdle A., McArdle F., Pattwell D.M., Bell G.M., Kemp G.J., Bone J.M., Griffiths R.D., Jackson M.J. (2007). Markers of oxidative stress in the skeletal muscle of patients on haemodialysis. Nephrol. Dial. Transplant..

[82] Ryan A.S. (2020). Role of Skeletal Muscle Mitochondrial Dysfunction in CKD. Clin. J. Am. Soc. Nephrol..

[83] Roshanravan B., Kestenbaum B., Gamboa J., Jubrias S.A., Ayers E., Curtin L., Himmelfarb J., de Boer I.H., Conley K.E. (2016). CKD and Muscle Mitochondrial Energetics. Am. J. Kidney Dis..

[84] Echtay K.S., Roussel D., St-Pierre J., Jekabsons M.B., Cadenas S., Stuart J.A., Harper J.A., Roebuck S.J., Morrison A., Pickering S. (2002). Superoxide activates mitochondrial uncoupling proteins. Nature.

[85] Gamboa J.L., Billings F.T., Bojanowski M.T., Gilliam L.A., Yu C., Roshanravan B., Roberts L.J., Himmelfarb J., Ikizler T.A., Brown N.J. (2016). Mitochondrial dysfunction and oxidative stress in patients with chronic kidney disease. Physiol. Rep..

[86] Gamboa J.L., Roshanravan B., Towse T., Keller C.A., Falck A.M., Yu C., Frontera W.R., Brown N.J., Ikizler T.A. (2020). Skeletal Muscle Mitochondrial Dysfunction Is Present in Patients with CKD Before Initiation of Maintenance Hemodialysis. Clin. J. Am. Soc. Nephrol..

[87] Siew E.D., Pupim L.B., Majchrzak K.M., Shintani A., Flakoll P.J., Ikizler T.A. (2007). Insulin resistance is associated with skeletal muscle protein breakdown in non-diabetic chronic hemodialysis patients. Kidney Int..

[88] Johansen K.L., Mulligan K., Schambelan M. (1999). Anabolic effects of nandrolone decanoate in patients receiving dialysis: A randomized controlled trial. JAMA.

[89] Cattran D.C., Fenton S.S., Wilson D.R., Oreopoulos D., Shimizu A., Richardson R.M. (1977). A controlled trial of nondrolone decanoate in the treatment of uremic anemia. Kidney Int..

[90] Chiang J.M., Kaysen G.A., Segal M., Chertow G.M., Delgado C., Johansen K.L. (2019). Low testosterone is associated with frailty, muscle wasting and physical dysfunction among men receiving hemodialysis: A longitudinal analysis. Nephrol. Dial. Transplant..

[91] Johansen K.L., Painter P.L., Sakkas G.K., Gordon P., Doyle J., Shubert T. (2006). Effects of resistance exercise training and nandrolone decanoate on body composition and muscle function among patients who receive hemodialysis: A randomized, controlled trial. J. Am. Soc. Nephrol..

[92] Badura K., Janc J., Wasik J., Gnitecki S., Skwira S., Mlynarska E., Rysz J., Franczyk B. (2024). Anemia of Chronic Kidney Disease—A Narrative Review of Its Pathophysiology, Diagnosis, and Management. Biomedicines.

[93] Davenport A., King R.F., Ironside J.W., Will E.J., Davison A.M. (1993). The effect of treatment with recombinant human erythropoietin on the histological appearance and glycogen content of skeletal muscle in patients with chronic renal failure treated by regular hospital haemodialysis. Nephron.

[94] Davenport A. (1993). The effect of treatment with recombinant human erythropoietin on skeletal muscle function in patients with end-stage renal failure treated with regular hospital hemodialysis. Am. J. Kidney Dis..

[95] Organ J.M., Srisuwananukorn A., Price P., Joll J.E., Biro K.C., Rupert J.E., Chen N.X., Avin K.G., Moe S.M., Allen M.R. (2016). Reduced skeletal muscle function is associated with decreased fiber cross-sectional area in the Cy/+ rat model of progressive kidney disease. Nephrol. Dial. Transplant..

[96] Fahal I.H., Bell G.M., Bone J.M., Edwards R.H. (1997). Physiological abnormalities of skeletal muscle in dialysis patients. Nephrol. Dial. Transplant..

[97] Johansen K.L., Shubert T., Doyle J., Soher B., Sakkas G.K., Kent-Braun J.A. (2003). Muscle atrophy in patients receiving hemodialysis: Effects on muscle strength, muscle quality, and physical function. Kidney Int..

[98] Bakinowska E., Olejnik-Wojciechowska J., Kielbowski K., Skoryk A., Pawlik A. (2024). Pathogenesis of Sarcopenia in Chronic Kidney Disease-The Role of Inflammation, Metabolic Dysregulation, Gut Dysbiosis, and microRNA. Int. J. Mol. Sci..

[99] Alston H., Burns A., Davenport A. (2018). Loss of appendicular muscle mass in haemodialysis patients is associated with increased self-reported depression, anxiety and lower general health scores. Nephrology.

[100] Xue Q.L. (2011). The frailty syndrome: Definition and natural history. Clin. Geriatr. Med..

[101] Kamijo Y., Kanda E., Ishibashi Y., Yoshida M. (2018). Sarcopenia and Frailty in PD: Impact on Mortality, Malnutrition, and Inflammation. Perit. Dial. Int..

[102] Abdala R., Elena Del Valle E., Negri A.L., Bridoux P., Paganti L.G., Bravo M., Sintado L., Di Rienzo P., Schiavelli O.R., Zanchetta M.B. (2021). Sarcopenia in hemodialysis patients from Buenos Aires, Argentina. Osteoporos. Sarcopenia.

[103] Cheng D., Zhang Q., Wang Z., Li J., Jian G., Wang N. (2021). Association Between Sarcopenia and Its Components and Dependency in Activities of Daily Living in Patients on Hemodialysis. J. Ren. Nutr..

[104] Giglio J., Kamimura M.A., Lamarca F., Rodrigues J., Santin F., Avesani C.M. (2018). Association of Sarcopenia with Nutritional Parameters, Quality of Life, Hospitalization, and Mortality Rates of Elderly Patients on Hemodialysis. J. Ren. Nutr..

[105] Lin Y.-L., Liou H.-H., Wang C.-H., Lai Y.-H., Kuo C.-H., Chen S.-Y., Hsu B.-G. (2020). Impact of sarcopenia and its diagnostic criteria on hospitalization and mortality in chronic hemodialysis patients: A 3-year longitudinal study. J. Formos. Med. Assoc..

[106] Bonanni A., Mannucci I., Verzola D., Sofia A., Saffioti S., Gianetta E., Garibotto G. (2011). Protein-energy wasting and mortality in chronic kidney disease. Int. J. Environ. Res. Public Health.

[107] Hanna R.M., Ghobry L., Wassef O., Rhee C.M., Kalantar-Zadeh K. (2020). A Practical Approach to Nutrition, Protein-Energy Wasting, Sarcopenia, and Cachexia in Patients with Chronic Kidney Disease. Blood Purif..

[108] Baltacı M.A., Atmis V., Metin Y., Aktar M., Eren S.A., Sengul S., Ates K., Kutlay S. (2023). Sarcopenia and cardiovascular risk indices: Its impact on cardiovascular events and mortality in dialysis patients. Semin. Dial..

[109] Hotta C., Hiraki K., Wakamiya A., Otobe Y., Watanabe S., Izawa K.P., Kaneshiro N., Konno Y., Sakurada T., Shibagaki Y. (2015). Relation of physical function and physical activity to sarcopenia in hemodialysis patients: A preliminary study. Int. J. Cardiol..

[110] Kim J.-K., Kim S.G., Oh J.-E., Lee Y.-K., Noh J.-W., Kim H.J., Song Y.R. (2019). Impact of sarcopenia on long-term mortality and cardiovascular events in patients undergoing hemodialysis. Korean J. Intern. Med..

[111] Li C., Chen L., He L., Zhang Y., Chen H., Liu Y., Tang S., Zheng H. (2022). Study on the relationship between sarcopenia and its components and anorexia in elderly maintenance haemodialysis patients. Nurs. Open.

[112] Elder M., Moonen A., Crowther S., Aleksova J., Center J., Elder G.J. (2023). Chronic kidney disease-related sarcopenia as a prognostic indicator in elderly haemodialysis patients. BMC Nephrol..

[113] Ferreira M.F., Böhlke M., Pauletto M.B., Frühauf I.R., Gonzalez M.C. (2022). Sarcopenia diagnosis using different criteria as a predictor of early mortality in patients undergoing hemodialysis. Nutrition.

[114] Lee H., Kim K., Ahn J., Lee D.R., Lee J.H., Hwang S.D. (2020). Association of nutritional status with osteoporosis, sarcopenia, and cognitive impairment in patients on hemodialysis. Asia Pac. J. Clin. Nutr..

[115] Hayashi H., Izumiya Y., Hayashi O., Ichii M., Tsujimoto Y., Yoshiyama M. (2022). Dynapenia is an independent predictor of cardio-cerebrovascular events in patients undergoing hemodialysis. Heart Vessels.

[116] Son H.E., Ryu J.Y., Lee K., Choi Y.I., Kim M.S., Park I., Shin G.T., Kim H., Ahn C., Kim S. (2022). The importance of muscle mass in predicting intradialytic hypotension in patients undergoing maintenance hemodialysis. Kidney Res. Clin. Pract..

[117] Ishimura E., Okuno S., Nakatani S., Mori K., Miyawaki J., Okazaki H., Sugie N., Norimine K., Yamakawa K., Tsujimoto Y. (2022). Significant Association of Diabetes with Mortality of Chronic Hemodialysis Patients, Independent of the Presence of Obesity, Sarcopenia, and Sarcopenic Obesity. J. Ren. Nutr..

[118] de Luca Corrêa H., Gadelha A.B., Vainshelboim B., Dutra M.T., Ferreira-Júnior J.B., Deus L.A., Neves R.V.P., Reis A.L., de Araújo T.B., Tzanno-Martins C. (2023). Could sarcopenia-related mortality in end-stage renal disease be underpinned by the number of hospitalizations and cardiovascular diseases?. Int. Urol. Nephrol..

[119] Kittiskulnam P., Chertow G.M., Carrero J.J., Delgado C., Kaysen G.A., Johansen K.L. (2017). Sarcopenia and its individual criteria are associated, in part, with mortality among patients on hemodialysis. Kidney Int..

[120] Kobayashi H., Takahashi M., Fukutomi M., Oba Y., Funayama H., Kario K. (2021). The long-term prognostic factors in hemodialysis patients with acute coronary syndrome: Perspectives from sarcopenia and malnutrition. Heart Vessels.

[121] Kono K., Moriyama Y., Yabe H., Hara A., Ishida T., Yamada T., Nishida Y. (2021). Relationship between malnutrition and possible sarcopenia in the AWGS 2019 consensus affecting mortality in hemodialysis patients: A prospective cohort study. BMC Nephrol..

[122] Mori K., Nishide K., Okuno S., Shoji T., Emoto M., Tsuda A., Nakatani S., Imanishi Y., Ishimura E., Yamakawa T. (2019). Impact of diabetes on sarcopenia and mortality in patients undergoing hemodialysis. BMC Nephrol..

[123] Sánchez-Tocino M.L., Miranda-Serrano B., López-González A., Villoria-González S., Pereira-García M., Gracia-Iguacel C., González-Ibarguren I., Ortíz-Arduan A., Mas-Fontao S., González-Parra E. (2022). Sarcopenia and Mortality in Older Hemodialysis Patients. Nutrients.

[124] Lin T.Y., Wu M.Y., Chen H.S., Hung S.C., Lim P.S. (2021). Development and validation of a multifrequency bioimpedance spectroscopy equation to predict appendicular skeletal muscle mass in hemodialysis patients. Clin. Nutr..

[125] Tsujimoto N., Matsuzawa R., Kakita D., Imai H., Harada M., Yoshikoshi S., Yamabe S., Osada S., Shimokado K., Matsunaga A. (2024). Concomitant sarcopenia and undernutrition: Impact on clinical outcomes in patients undergoing hemodialysis. Clin. Nutr. ESPEN.

[126] Ulgen C., Ozturk I., Sahin M., Guzel F.B., Oguz A., Altunoren O., Gungor O. (2023). The amount of skeletal muscle mass is associated with arterial stiffness in hemodialysis patients. Ther. Apher. Dial..

[127] Wang Y., Ma W., Pu J., Chen F. (2023). Interrelationships between sarcopenia, bone turnover markers and low bone mineral density in patients on hemodialysis. Ren. Fail..

[128] Xavier J.S., Góes C.R., Borges M.C.C., Caramori J.C.T., Vogt B.P. (2022). Handgrip Strength Thresholds are Associated with Malnutrition Inflammation Score (MIS) in Maintenance Hemodialysis Patients. J. Ren. Nutr..

[129] Xiang T., Fu P., Zhou L. (2023). Sarcopenia and osteosarcopenia among patients undergoing hemodialysis. Front. Endocrinol..

[130] Yang Y., Da J., Yuan J., Zha Y. (2023). One-year change in sarcopenia was associated with cognitive impairment among haemodialysis patients. J. Cachexia Sarcopenia Muscle.

[131] Yoshikoshi S., Yamamoto S., Suzuki Y., Imamura K., Harada M., Kamiya K., Matsunaga A. (2024). Prevalence of osteosarcopenia and its association with mortality and fractures among patients undergoing hemodialysis. J. Bone Miner. Metab..

[132] Yoshikoshi S., Yamamoto S., Suzuki Y., Imamura K., Harada M., Osada S., Kamiya K., Matsunaga A. (2022). Associations between dynapenia, cardiovascular hospitalizations, and all-cause mortality among patients on haemodialysis. J. Cachexia Sarcopenia Muscle.

[133] Lee Y.H., Kim J.S., Jung S.W., Hwang H.S., Moon J.Y., Jeong K.H., Lee S.H., Lee S.Y., Ko G.J., Lee D.Y. (2020). Gait speed and handgrip strength as predictors of all-cause mortality and cardiovascular events in hemodialysis patients. BMC Nephrol..

[134] Yuenyongchaiwat K., Jongritthiporn S., Somsamarn K., Sukkho O., Pairojkittrakul S., Traitanon O. (2021). Depression and low physical activity are related to sarcopenia in hemodialysis: A single-center study. PeerJ.

[135] Zhou C., Zhan L., He P., Yuan J., Zha Y. (2023). Associations of sarcopenic obesity vs. either sarcopenia or obesity alone with cognitive impairment risk in patients requiring maintenance hemodialysis. Nutr. Clin. Pract..

[136] Lin Y.L., Hsu B.G. (2022). Assessment of uremic sarcopenia in dialysis patients: An update. Tzu Chi Med. J..

[137] Giraudo C., Cavaliere A., Lupi A., Guglielmi G., Quaia E. (2020). Established paths and new avenues: A review of the main radiological techniques for investigating sarcopenia. Quant. Imaging Med. Surg..

[138] Studenski S.A., Peters K.W., Alley D.E., Cawthon P.M., McLean R.R., Harris T.B., Ferrucci L., Guralnik J.M., Fragala M.S., Kenny A.M. (2014). The FNIH sarcopenia project: Rationale, study description, conference recommendations, and final estimates. J. Gerontol. Ser. A Biomed. Sci. Med. Sci..

[139] Cruz-Jentoft A.J., Bahat G., Bauer J., Boirie Y., Bruyere O., Cederholm T., Cooper C., Landi F., Rolland Y., Sayer A.A. (2019). Sarcopenia: Revised European consensus on definition and diagnosis. Age Ageing.

[140] Chen L.K., Woo J., Assantachai P., Auyeung T.W., Chou M.Y., Iijima K., Jang H.C., Kang L., Kim M., Kim S. (2020). Asian Working Group for Sarcopenia: 2019 Consensus Update on Sarcopenia Diagnosis and Treatment. J. Am. Med. Dir. Assoc..

[141] Fielding R.A., Vellas B., Evans W.J., Bhasin S., Morley J.E., Newman A.B., Abellan van Kan G., Andrieu S., Bauer J., Breuille D. (2011). Sarcopenia: An undiagnosed condition in older adults. Current consensus definition: Prevalence, etiology, and consequences. International working group on sarcopenia. J. Am. Med. Dir. Assoc..

[142] Chamney P.W., Wabel P., Moissl U.M., Muller M.J., Bosy-Westphal A., Korth O., Fuller N.J. (2007). A whole-body model to distinguish excess fluid from the hydration of major body tissues. Am. J. Clin. Nutr..

[143] Wabel P., Chamney P., Moissl U., Jirka T. (2009). Importance of whole-body bioimpedance spectroscopy for the management of fluid balance. Blood Purif..

[144] Reis N., Vaninni F.C.D., Silva M.Z.C., de Oliveira R.C., Reis F.M., Costa F.L., Martin L.C., Barretti P. (2021). Agreement of Single-Frequency Electrical Bioimpedance in the Evaluation of Fat Free Mass and Fat Mass in Peritoneal Dialysis Patients. Front. Nutr..

[145] Bellafronte N.T., Batistuti M.R., Dos Santos N.Z., Holland H., Romão E.A., Chiarello P.G. (2018). Estimation of Body Composition and Water Data Depends on the Bioelectrical Impedance Device. J. Electr. Bioimpedance.

[146] Kim C., Kim J.K., Lee H.S., Kim S.G., Song Y.R. (2021). Longitudinal changes in body composition are associated with all-cause mortality in patients on peritoneal dialysis. Clin. Nutr..

[147] Nijholt W., Scafoglieri A., Jager-Wittenaar H., Hobbelen J.S.M., van der Schans C.P. (2017). The reliability and validity of ultrasound to quantify muscles in older adults: A systematic review. J. Cachexia Sarcopenia Muscle.

[148] Perkisas S., Baudry S., Bauer J., Beckwee D., De Cock A.M., Hobbelen H., Jager-Wittenaar H., Kasiukiewicz A., Landi F., Marco E. (2018). Application of ultrasound for muscle assessment in sarcopenia: Towards standardized measurements. Eur. Geriatr. Med..

[149] Matsuzawa R., Yamamoto S., Suzuki Y., Imamura K., Harada M., Matsunaga A., Tamaki A., Fukui T., Shimokado K. (2021). The clinical applicability of ultrasound technique for diagnosis of sarcopenia in hemodialysis patients. Clin. Nutr..

[150] Sabatino A., Kooman J.P., Di Motta T., Cantarelli C., Gregorini M., Bianchi S., Regolisti G., Fiaccadori E. (2022). Quadriceps muscle thickness assessed by ultrasound is independently associated with mortality in hemodialysis patients. Eur. J. Clin. Nutr..

[151] Sabatino A., Kooman J., Avesani C.M., Gregorini M., Bianchi S., Regolisti G., Fiaccadori E. (2024). Sarcopenia diagnosed by ultrasound-assessed quadriceps muscle thickness and handgrip strength predicts mortality in patients on hemodialysis. J. Nephrol..

[152] MacRae J.M., Harasemiw O., Lightfoot C.J., Thompson S., Wytsma-Fisher K., Koufaki P., Bohm C., Wilkinson T.J. (2023). Measurement properties of performance-based measures to assess physical function in chronic kidney disease: Recommendations from a COSMIN systematic review. Clin. Kidney J..

[153] Pinto A.P., Ramos C.I., Meireles M.S., Kamimura M.A., Cuppari L. (2015). Impact of hemodialysis session on handgrip strength. J. Bras. Nefrol..

[154] Fahal I.H. (2014). Uraemic sarcopenia: Aetiology and implications. Nephrol. Dial. Transplant..

[155] Bataille S., Serveaux M., Carreno E., Pedinielli N., Darmon P., Robert A. (2017). The diagnosis of sarcopenia is mainly driven by muscle mass in hemodialysis patients. Clin. Nutr..

[156] Xu X., Yang Z., Ma T., Li Z., Chen Y., Zheng Y., Dong J. (2020). The cut-off values of handgrip strength and lean mass index for sarcopenia among patients on peritoneal dialysis. Nutr. Metab..

[157] Vogt B.P., Borges M.C.C., Goes C.R., Caramori J.C.T. (2016). Handgrip strength is an independent predictor of all-cause mortality in maintenance dialysis patients. Clin. Nutr..

[158] Vanden Wyngaert K., Van Biesen W., Eloot S., Van Craenenbroeck A.H., Calders P., Holvoet E. (2022). The importance of physical performance in the assessment of patients on haemodialysis: A survival analysis. PLoS ONE.

[159] Duarte M.P., Nobrega O.T., Vogt B.P., Pereira M.S., Silva M.Z.C., Mondini D.R., Disessa H.S., Adamoli A.N., Bundchen D.C., Sant’Helena B.R.M. (2024). Reference Values for Handgrip Strength, Five Times Sit-to-Stand, and Gait Speed in Patients on Hemodialysis. Nephrol. Dial. Transplant..

[160] Malmstrom T.K., Morley J.E. (2013). SARC-F: A simple questionnaire to rapidly diagnose sarcopenia. J. Am. Med. Dir. Assoc..

[161] Lin Y.L., Hou J.S., Lai Y.H., Wang C.H., Kuo C.H., Liou H.H., Hsu B.G. (2020). Association of SARC-F Questionnaire and Mortality in Prevalent Hemodialysis Patients. Diagnostics.

[162] Barbosa-Silva T.G., Menezes A.M., Bielemann R.M., Malmstrom T.K., Gonzalez M.C., Grupo de Estudos em Composição Corporal e Nutrição (COCONUT) (2016). Enhancing SARC-F: Improving Sarcopenia Screening in the Clinical Practice. J. Am. Med. Dir. Assoc..

[163] Lin Y.L., Wang C.H., Tsai J.P., Chen C.T., Chen Y.H., Hung S.C., Hsu B.G. (2022). A Comparison of SARC-F, Calf Circumference, and Their Combination for Sarcopenia Screening Among Patients Undergoing Peritoneal Dialysis. Nutrients.

[164] Kakita D., Matsuzawa R., Yamamoto S., Suzuki Y., Harada M., Imamura K., Yoshikoshi S., Imai H., Osada S., Shimokado K. (2022). Simplified discriminant parameters for sarcopenia among patients undergoing haemodialysis. J. Cachexia Sarcopenia Muscle.

[165] Canaud B., Ye X., Usvyat L., Kooman J., van der Sande F., Raimann J., Wang Y., Kotanko P. (2020). Clinical and predictive value of simplified creatinine index used as muscle mass surrogate in end-stage kidney disease haemodialysis patients-results from the international MONitoring Dialysis Outcome initiative. Nephrol. Dial. Transplant..

[166] Lee J.H., Jun H.S. (2019). Role of Myokines in Regulating Skeletal Muscle Mass and Function. Front. Physiol..

[167] Verzola D., Barisione C., Picciotto D., Garibotto G., Koppe L. (2019). Emerging role of myostatin and its inhibition in the setting of chronic kidney disease. Kidney Int..

[168] Zhou Y., Hellberg M., Hellmark T., Hoglund P., Clyne N. (2021). Muscle mass and plasma myostatin after exercise training: A substudy of Renal Exercise (RENEXC)-a randomized controlled trial. Nephrol. Dial. Transplant..

[169] Sakashita M., Hamasaki Y., Oki R., Komaru Y., Miyamoto Y., Yoshida T., Matsuura R., Doi K., Nangaku M. (2024). Serum Myostatin at Dialysis Initiation May Predict 1-Year Mortality and Hospitalization. Nephron.

[170] Qaisar R., Karim A., Muhammad T., Shah I., Khan J. (2021). Prediction of sarcopenia using a battery of circulating biomarkers. Sci. Rep..

[171] Kwak J.Y., Hwang H., Kim S.K., Choi J.Y., Lee S.M., Bang H., Kwon E.S., Lee K.P., Chung S.G., Kwon K.S. (2018). Prediction of sarcopenia using a combination of multiple serum biomarkers. Sci. Rep..

[172] Park J., Mehrotra R., Rhee C.M., Molnar M.Z., Lukowsky L.R., Patel S.S., Nissenson A.R., Kopple J.D., Kovesdy C.P., Kalantar-Zadeh K. (2013). Serum creatinine level, a surrogate of muscle mass, predicts mortality in peritoneal dialysis patients. Nephrol. Dial. Transplant..

[173] Kalantar-Zadeh K., Streja E., Kovesdy C.P., Oreopoulos A., Noori N., Jing J., Nissenson A.R., Krishnan M., Kopple J.D., Mehrotra R. (2010). The obesity paradox and mortality associated with surrogates of body size and muscle mass in patients receiving hemodialysis. Mayo Clin. Proc..

[174] Keshaviah P.R., Nolph K.D., Moore H.L., Prowant B., Emerson P.F., Meyer M., Twardowski Z.J., Khanna R., Ponferrada L., Collins A. (1994). Lean body mass estimation by creatinine kinetics. J. Am. Soc. Nephrol..

[175] Noori N., Kovesdy C.P., Bross R., Lee M., Oreopoulos A., Benner D., Mehrotra R., Kopple J.D., Kalantar-Zadeh K. (2011). Novel equations to estimate lean body mass in maintenance hemodialysis patients. Am. J. Kidney Dis..

[176] Zhang F., Yin X., Huang L., Zhang H. (2023). The “adult inactivity triad” in patients with chronic kidney disease: A review. Front. Med..

[177] Battaglia Y., Baciga F., Bulighin F., Amicone M., Mosconi G., Storari A., Brugnano R., Pozzato M., Motta D., D’Alessandro C. (2024). Physical activity and exercise in chronic kidney disease: Consensus statements from the Physical Exercise Working Group of the Italian Society of Nephrology. J. Nephrol..

[178] Deligiannis A., D’Alessandro C., Cupisti A. (2021). Exercise training in dialysis patients: Impact on cardiovascular and skeletal muscle health. Clin. Kidney J..

[179] Araujo A.M., Orcy R.B., Feter N., Weymar M.K., Cardoso R.K., Bohlke M., Rombaldi A.J. (2024). Effects of intradialytic exercise on functional capacity in patients with end-stage chronic kidney disease: A systematic review and meta-analysis. Res. Sports Med..

[180] Sabatino A., Cuppari L., Stenvinkel P., Lindholm B., Avesani C.M. (2021). Sarcopenia in chronic kidney disease: What have we learned so far?. J. Nephrol..

[181] Kopple J.D., Wang H., Casaburi R., Fournier M., Lewis M.I., Taylor W., Storer T.W. (2007). Exercise in maintenance hemodialysis patients induces transcriptional changes in genes favoring anabolic muscle. J. Am. Soc. Nephrol..

[182] Bulighin F., Aucella F., Bellizzi V., Cupisti A., Faga T., Gambaro G., Regolisti G., Storari A., Capitanini A., Battaglia Y. (2024). Physical activity and exercise programs for kidney patients: An Italian survey of nephrology centres. J. Nephrol..

[183] Kidney Disease: Improving Global Outcomes (KDIGO) CKD Work Group (2024). KDIGO 2024 Clinical Practice Guideline for the Evaluation and Management of Chronic Kidney Disease. Kidney Int..

[184] Bellizzi V., Regolisti G. (2022). What is the role of exercise in chronic kidney disease?. Nephrol. Dial. Transplant..

[185] Clyne N., Anding-Rost K. (2021). Exercise training in chronic kidney disease-effects, expectations and adherence. Clin. Kidney J..

[186] Lambert K., Lightfoot C.J., Jegatheesan D.K., Gabrys I., Bennett P.N. (2022). Physical activity and exercise recommendations for people receiving dialysis: A scoping review. PLoS ONE.

[187] Kirkman D.L., Mullins P., Junglee N.A., Kumwenda M., Jibani M.M., Macdonald J.H. (2014). Anabolic exercise in haemodialysis patients: A randomised controlled pilot study. J. Cachexia Sarcopenia Muscle.

[188] Koufaki P., Greenwood S., Painter P., Mercer T. (2015). The BASES expert statement on exercise therapy for people with chronic kidney disease. J. Sports Sci..

[189] Baggetta R., Bolignano D., Torino C., Manfredini F., Aucella F., Barilla A., Battaglia Y., Bertoli S., Bonanno G., Castellino P. (2014). Fitness for entering a simple exercise program and mortality: A study corollary to the exercise introduction to enhance performance in dialysis (EXCITE) trial. Kidney Blood Press. Res..

[190] Baggetta R., D’Arrigo G., Torino C., ElHafeez S.A., Manfredini F., Mallamaci F., Zoccali C., Tripepi G., EXCITE Working group (2018). Effect of a home based, low intensity, physical exercise program in older adults dialysis patients: A secondary analysis of the EXCITE trial. BMC Geriatr..

[191] Manfredini F., Mallamaci F., D’Arrigo G., Baggetta R., Bolignano D., Torino C., Lamberti N., Bertoli S., Ciurlino D., Rocca-Rey L. (2017). Exercise in Patients on Dialysis: A Multicenter, Randomized Clinical Trial. J. Am. Soc. Nephrol..

[192] Manfredini F., D’Arrigo G., Lamberti N., Torino C., Tripepi G., Mallamaci F., Zoccali C. (2022). The legacy effect of a home walking exercise programme in kidney failure patients on dialysis. Nephrol. Dial. Transplant..

[193] Baker L.A., March D.S., Wilkinson T.J., Billany R.E., Bishop N.C., Castle E.M., Chilcot J., Davies M.D., Graham-Brown M.P.M., Greenwood S.A. (2022). Clinical practice guideline exercise and lifestyle in chronic kidney disease. BMC Nephrol..

[194] Liu Y., Luo X., Deng S., Chen J., Zhang L., Huang Y., Hu H. (2023). Combined aerobic and resistance exercise in maintenance hemodialysis patients: A meta-analysis. Semin. Dial..

[195] Yin J., Zhang X., Wang Z., Qu Z., Sun X., Song Y., Zhang H. (2024). Application of exercise therapy in patients with chronic kidney disease-induced muscle atrophy: A scoping review. BMC Sports Sci. Med. Rehabil..

[196] Battaglia Y., Amicone M., Mantovani A., Combe C., Mitra S., Basile C., EuDial Working Group of ERA (2023). Home-based exercise in patients on maintenance dialysis: A systematic review and meta-analysis of randomized clinical trials. Nephrol. Dial. Transplant..

[197] Sabatino A., Piotti G., Cosola C., Gandolfini I., Kooman J.P., Fiaccadori E. (2018). Dietary protein and nutritional supplements in conventional hemodialysis. Semin. Dial..

[198] Matsuzawa R., Yamamoto S., Suzuki Y., Abe Y., Harada M., Shimoda T., Imamura K., Yamabe S., Ito H., Yoshikoshi S. (2021). The effects of amino acid/protein supplementation in patients undergoing hemodialysis: A systematic review and meta-analysis of randomized controlled trials. Clin. Nutr. ESPEN.

[199] Mori K. (2021). Maintenance of Skeletal Muscle to Counteract Sarcopenia in Patients with Advanced Chronic Kidney Disease and Especially Those Undergoing Hemodialysis. Nutrients.

[200] Molina P., Carrero J.J., Bover J., Chauveau P., Mazzaferro S., Torres P.U., European Renal Nutrition (ERN) and Chronic Kidney Disease-Mineral and Bone Disorder (CKD-MBD) Working Groups of the European Renal Association—European Dialysis Transplant Association (ERA-EDTA) (2017). Vitamin D, a modulator of musculoskeletal health in chronic kidney disease. J. Cachexia Sarcopenia Muscle.

[201] Marckmann P., Agerskov H., Thineshkumar S., Bladbjerg E.M., Sidelmann J.J., Jespersen J., Nybo M., Rasmussen L.M., Hansen D., Scholze A. (2012). Randomized controlled trial of cholecalciferol supplementation in chronic kidney disease patients with hypovitaminosis D. Nephrol. Dial. Transplant..

[202] Hewitt N.A., O’Connor A.A., O’Shaughnessy D.V., Elder G.J. (2013). Effects of cholecalciferol on functional, biochemical, vascular, and quality of life outcomes in hemodialysis patients. Clin. J. Am. Soc. Nephrol..

[203] Gordon P.L., Sakkas G.K., Doyle J.W., Shubert T., Johansen K.L. (2007). Relationship between vitamin D and muscle size and strength in patients on hemodialysis. J. Ren. Nutr..

[204] Kang S.H., Do J.Y., Cho J.H., Jeong H.Y., Yang D.H., Kim J.C. (2020). Association Between Vitamin D Level and Muscle Strength in Patients Undergoing Hemodialysis. Kidney Blood Press. Res..

[205] Lin Y.L., Chen S.Y., Lai Y.H., Wang C.H., Kuo C.H., Liou H.H., Hsu B.G. (2019). Angiotensin II receptor blockade is associated with preserved muscle strength in chronic hemodialysis patients. BMC Nephrol..

[206] Picciotto D., Maccio L., Verzola D., Baciga F., Momente C., Russo E., Viazzi F., Battaglia Y., Esposito P. (2024). Pathophysiology of Physical Exercise in Kidney Patients: Unveiling New Players—The Role of Myokines. Kidney Blood Press. Res..

[207] Esposito P., Picciotto D., Battaglia Y., Costigliolo F., Viazzi F., Verzola D. (2022). Myostatin: Basic biology to clinical application. Adv. Clin. Chem..

[208] Esposito P., Battaglia Y., La Porta E., Grignano M.A., Caramella E., Avella A., Peressini S., Sessa N., Albertini R., Di Natali G. (2019). Significance of serum Myostatin in hemodialysis patients. BMC Nephrol..

[209] Esposito P., La Porta E., Calatroni M., Grignano M.A., Milanesi S., Verzola D., Battaglia Y., Gregorini M., Libetta C., Garibotto G. (2017). Modulation of Myostatin/Hepatocyte Growth Factor Balance by Different Hemodialysis Modalities. BioMed Res. Int..

[210] Bataille S., Chauveau P., Fouque D., Aparicio M., Koppe L. (2021). Myostatin and muscle atrophy during chronic kidney disease. Nephrol. Dial. Transplant..

[211] Gascon A., Belvis J.J., Berisa F., Iglesias E., Estopinan V., Teruel J.L. (1999). Nandrolone decanoate is a good alternative for the treatment of anemia in elderly male patients on hemodialysis. Geriatr. Nephrol. Urol..

[212] Macdonald J.H., Marcora S.M., Jibani M.M., Kumwenda M.J., Ahmed W., Lemmey A.B. (2007). Nandrolone decanoate as anabolic therapy in chronic kidney disease: A randomized phase II dose-finding study. Nephron Clin. Pract..

[213] Noce A., Marrone G., Ottaviani E., Guerriero C., Di Daniele F., Pietroboni Zaitseva A., Di Daniele N. (2021). Uremic Sarcopenia and Its Possible Nutritional Approach. Nutrients.

